# Premature mortality in people affected by co-occurring homelessness, justice involvement, opioid dependence, and psychosis: a retrospective cohort study using linked administrative data

**DOI:** 10.1016/S2468-2667(22)00159-1

**Published:** 2022-07-28

**Authors:** Emily J Tweed, Alastair H Leyland, David Morrison, S Vittal Katikireddi

**Affiliations:** a MRC/CSO Social and Public Health Sciences Unit, University of Glasgow, Glasgow, UK; b Institute of Health and Wellbeing, University of Glasgow, Glasgow, UK

## Abstract

**Background:**

Homelessness, opioid dependence, justice involvement, and psychosis are each associated with an increased risk of poor health and commonly co-occur in the same individuals. Most existing studies of mortality associated with this co-occurrence rely on active follow-up methods prone to selection and retention bias, and focus on a limited set of specific exposures rather than taking a population-based approach. To address these limitations, we did a retrospective cohort study using linked administrative data.

**Methods:**

In this retrospective cohort study, we linked a population register of adults resident in Glasgow, UK, to administrative datasets from homelessness and criminal justice services; community pharmacies; and a clinical psychosis registry with data from April 1, 2010 to March 31, 2014. Linkage to death registrations from April 1, 2014 to March 31, 2019 provided follow-up data on premature mortality (age <75 years) from all causes, non-communicable diseases, and causes considered potentially avoidable through health-care or public health intervention. We estimated hazard ratios (HR) using Poisson regression, adjusting for age, gender, socioeconomic deprivation, and calendar time.

**Findings:**

Of 536 653 cohort members, 11 484 (2·1%) died during follow-up. All-cause premature mortality was significantly higher among people with multiple exposures than among people with single exposures, and among people with any exposure than among people with none (eg, homelessness plus other exposures *vs* no exposures: HR 8·4 [95% CI 7·3–9·5]; homelessness alone *vs* no exposures: HR 2·2 [1·9–2·5]). Avoidable premature mortality was highest among those with multiple exposures (eg, imprisonment plus other exposures *vs* no exposures: HR 10·5 [9·1–12·3]; imprisonment alone *vs* no exposures: HR 3·8 [3·0–4·8]). Premature mortality from non-communicable disease was higher among those with any exposures than among those with none, despite accounting for a lower proportion of deaths in the exposed group; although in some cases there was little difference between estimates for single versus multiple exposures.

**Interpretation:**

The co-occurrence of at least two of homelessness, opioid dependence, justice involvement, or psychosis is associated with very high rates of premature mortality, particularly from avoidable causes of death, including non-communicable disease. Responding to these findings demands wide-ranging efforts across health-care provision, public health, and social policy. Future work should examine the timing and sequencing of exposures to better understand the causal pathways underlying excess mortality.

**Funding:**

Chief Scientist Office, Medical Research Council, NHS Research Scotland.

## Introduction

Experiences of homelessness, justice involvement, opioid dependence, and psychosis are characterised by exclusionary processes such as stigma and discrimination; restrictions on basic freedoms or rights (eg, voting, privacy, and liberty); or barriers to accessing public services (eg, health care).^1^, ^2^, ^3^, ^4^

Experiencing any one of these in isolation is associated with higher rates of ill health and premature death compared with unaffected peers, even after accounting for socioeconomic position.^5^ Evidence suggests that these experiences frequently co-occur, although the extent of this overlap varies by context.^6^, ^7^, ^8^ These experiences and their co-occurrence might influence health through multiple and complex pathways. For instance, their harmful effects might combine (or even synergise) or have no additional impact over the baseline risk conferred by each independently. Alternatively, multiple disadvantages might have a paradoxical beneficial effect by conferring additional entitlements or access to services (eg, where based on a threshold of need).

**Research in context**

**Evidence before this study**

We did a systematic review of morbidity and mortality among people with overlapping experiences of homelessness, substance use, imprisonment, severe mental illness, or sex work. We did a further search of MEDLINE, Embase, and PsycINFO on Dec 14, 2021, to identify more recent studies published in English since June 11, 2018 (the limit of the previous searches) and extend the scope to include people in contact with community justice services. We found that there was a scarcity of evidence on the health of people affected by these co-occurring experiences from longitudinal studies that used population-based samples, included multiple exposure combinations, and examined outcomes other than infectious disease, mental illness, and external causes (such as overdose, accidents, and assault). Evidence on mortality from non-communicable diseases and other potentially avoidable causes was especially scarce.

**Added value of this study**

This study draws on a novel, population-based cohort of more than half a million people, created by linking records between administrative datasets from multiple sectors. The study shows that much of the extremely high rate of premature mortality among people with co-occurring experiences of homelessness, justice involvement, opioid dependence, or psychosis is accounted for by causes that are avoidable through timely access to high-quality health-care and public health interventions, including a substantial burden of non-communicable diseases.

**Implications of all the available evidence**

Experiences of homelessness, justice involvement, opioid dependence, and psychosis commonly co-occur. In many countries, the population of people affected by these experiences is growing, and growing older. The intersection between these experiences is associated with extremely poor health outcomes, yet there appears to be substantial scope for prevention and mitigation through health-care and public health services. To date, service and policy responses have often been fragmented and uncoordinated, focusing on single issues in isolation and on a narrow range of health conditions. Efforts by health systems and other policy sectors to address these experiences must be reoriented to recognise intersecting forms of disadvantage and to better reflect those conditions causing the greatest burden of ill health.

A recent systematic review suggested that the co-occurrence of these experiences is associated with especially poor outcomes, but it identified very little evidence for conditions other than infections or external causes of morbidity and mortality (such as overdose, accidents, or assault), with particular gaps around the burden of non-communicable diseases and conditions avoidable through health-care or public health interventions.^9^ There was also a scarcity of longitudinal studies from countries outside North America, Scandinavia, and Australia and studies on exposure combinations other than imprisonment and substance use, or severe mental illness and substance use.

An accurate understanding of the burden of ill health in people with these experiences is essential to inform the development and implementation of services and policies that meet their needs and tackle inequalities in health. For instance, in the UK, the National Institute of Health and Care Excellence has highlighted a scarcity of evidence on the physical health needs of people with coexisting substance misuse and severe mental illness, and the mental health of adults in contact with the justice system.^10^, ^11^ Descriptive epidemiology can also provide a baseline picture against which efforts to address these forms of adversity—and their health consequences—can be evaluated.

One approach to this challenge is the use of administrative data, produced by services as a by-product of their day-to-day operations.^12^ Administrative data typically provide extensive (or even complete) population coverage; are of low cost to obtain; and have high external validity and policy relevance. Record linkage between such datasets across different sectors can be uniquely powerful in helping understand the social and structural determinants of health and identify opportunities for intervention on cross-cutting policy issues.^13^ This method is especially valuable in understanding the experiences and needs of marginalised populations who might be poorly represented in primary research, for instance due to ascertainment difficulties or participation burdens that affect recruitment and retention, but who often have high levels of need for and use of public services.

In this study, we aimed to use cross-sectoral linkage of administrative data to investigate premature mortality among a population cohort containing data on exposure to homelessness, justice involvement, opioid dependence, and psychosis, with a particular focus on mortality from potentially avoidable causes, mortality from non-communicable diseases, and years of potential life lost.

## Methods

### Study design and participants

In this retrospective cohort study, we used cross-sectoral record linkage of administrative datasets from local authorities, health-care services, and death registrations from the Glasgow City local authority area from April 1, 2010 to March 31, 2019. Glasgow had a population of 595 070 in 2012 (the mid-point of the exposure period for our primary analyses), accounting for 11% of the population of Scotland. The definitions of exposure and follow-up periods were determined by the availability and quality of the datasets of interest, which varied over time. For the study population, we identified a cohort of adults resident in the Glasgow City Council area using postcode of residence recorded within the Community Health Index (CHI; a unique ten-digit personal identifier used across the health service in Scotland) population register held by the West of Scotland Safe Haven secure data repository (appendix p 1). This dataset is derived from general practitioner registrations and is widely used in record linkage studies as a proxy for total population. We used administrative datasets to assign exposure status to individuals within the population cohort during the period of April 1, 2010 to March 31, 2014 (the exposure period; table 1). We excluded individuals recorded as having died or transferred out during the exposure period and individuals aged younger than 18 years at the start of the exposure period (given statutory age limitations on some of the services represented in the datasets). Given historical limitations on the availability of electronic death records for older individuals within the Safe Haven, and the low prevalence of these experiences in older age groups, we restricted analyses to individuals aged younger than 75 years at the start of follow-up and censored follow-up if participants turned 75 years of age during the study period (appendix pp 1–4).

**Table 1:** Data sources used in cohort creation to ascertain exposures

	**Definition**	**Data source**	**Data collection**	**Selection process (if any)**	**Data provider**
Homelessness or housing insecurity	Resident of Glasgow assessed by Glasgow City Council as homeless or threatened with homelessness (main applicant only)	HL1 dataset	Face-to-face interview between applicant and housing officer	Individual experiencing homelessness applies to local authority for support	Glasgow City Council
Justice involvement (any prison record)	Resident of Glasgow having previously been received into a Scottish prison	PR2 dataset	Reception process when individual arrives at prison	None	Scottish Prison Service and Scottish Government
Justice involvement (court report only)	Resident of Glasgow having been the subject of a submitted Criminal Justice Social Work Report	Criminal Justice Social Work Reports	Face-to-face interview between applicant and social work officer	Individual convicted of offence meets statutory criteria for Criminal Justice Social Work Report or request otherwise made by sheriff	Glasgow City Council
Opioid dependence	Resident of Glasgow having received community-dispensed opioid substitution therapy anywhere covered by NHS Greater Glasgow and Clyde	Prescribing Information System	Electronic record of dispensing, generated for reimbursement purposes	Individual with opioid dependence seeks treatment; is prescribed opioid substitution therapy; and redeems prescription	NHS Greater Glasgow and Clyde
Psychosis	Resident of Glasgow with diagnosis of psychotic disorder	Glasgow Psychosis Clinical Information System	Review of clinical records by research nurse, with or without correspondence with clinical team	Individual experiencing psychosis is in contact with community mental health team	NHS Greater Glasgow and Clyde

NHS=National Health Service.

Permission to access and link the relevant datasets was provided by the following organisations: the Local Privacy Advisory Committee of the West of Scotland Safe Haven (National Health Service [NHS] Greater Glasgow and Clyde population register, prescribing records, Psychosis Clinical Information System register, and death records); the Data Protection Officer and relevant Head of Service of Glasgow City Health and Social Care Partnership (HL1 and Criminal Justice Social Work Report datasets); and the Scottish Government Statistics Public Benefit and Privacy Panel and the Scottish Prison Service Research Access and Ethics Committee (PR2 dataset). Following approval from these organisations, a letter of comfort was issued by the research ethics committee of the University of Glasgow College of Medical, Veterinary, and Life Sciences. To minimise the risk of potential identification of individuals by deductive disclosure, on some occasions categories have been combined or results suppressed.

### Procedures

We defined each exposure as the presence of at least one episode in the relevant dataset during this 4-year period: as such, exposure definitions reflect the cumulative experience across this period. For justice involvement, we assigned individuals to one of two exposure categories using the combination of prison and court records: custodial (ie, any imprisonment during the study period, regardless of whether a court report was made) and community (ie, court report without imprisonment). Those without any episodes recorded in the administrative datasets used for exposure ascertainment were classified as unexposed.

To ensure sufficient size in each exposure group, and in light of our interest in premature mortality associated with multiple co-occurring exposures, the primary exposure categories used in mortality analyses classified exposed individuals into those with a given exposure in isolation (eg, homelessness alone) versus that exposure in combination with others (eg, homelessness plus opioid dependence), on the basis of their cumulative history during the exposure period.

Data on deaths among the cohort were obtained from death registrations collected by National Records of Scotland and provided to the West of Scotland Safe Haven. The follow-up period for mortality outcomes was defined as April 1, 2014 to March 31, 2019, with follow-up ceasing (ie, censoring) on the date of the earliest of the following four events: death; migration out of the area covered by NHS Greater Glasgow and Clyde (the area in which migration and mortality data were available); turning 75 years of age; or end of follow-up on March 31, 2019.

To account for the possibility of death on the first day of follow-up, 0·5 days of survival time were added for everyone in the cohort, except for those who did not die, migrate out, or turn age 75 years during the study period and therefore completed the full 1825 days of follow-up.

All record linkage was undertaken by the West of Scotland Safe Haven, with no personal identifiable information available to the research team at any stage. Those datasets originating with the NHS that did not already contain CHI numbers for all records underwent CHI assignment based on the population register using a deterministic method supplemented by manual review on the basis of forename (or forename Soundex code, an alphanumeric code used to anonymously represent similar-sounding surnames with different spellings), surname (or surname Soundex code), date of birth, and (for manual reviews only) postcode. All datasets were then linked deterministically using the CHI number, with a de-identified dataset being made available to the research team for analysis via a secure analytic environment within the Safe Haven (appendix pp 1–4).

### Outcomes

All-cause premature mortality was defined as death during follow-up from any cause before the age of 75 years, as per the definition applied by National Records of Scotland. Cause-specific mortality definitions were based on the underlying cause of death field, classified using codes from the International Classification of Diseases, version 10 (ICD-10). On the basis of evidence gaps identified in a previous systematic review, we focused on deaths from avoidable causes and from non-communicable diseases.^9^

Avoidable mortality is a widely used metric of health-care and public health system performance:^14^, ^15^ we used the internationally harmonised Organisation for Economic Co-operation and Development (OECD)-Eurostat 2019 definition, which further subdivides avoidable causes into preventable causes (those which can be mainly avoided by effective public health and primary prevention activity, such as deaths from vaccine-preventable diseases or skin cancer) and treatable causes (those which can be mainly avoided through timely access to high-quality health care, such as deaths from appendicitis or asthma).^16^ For non-communicable disease mortality, we used the NCD4 definition employed by the WHO NCD Global Monitoring Framework and UN Sustainable Development Goals, comprising four main types of non-communicable disease: cancer, cardiovascular disease, diabetes, and chronic respiratory disease.^17^ ICD-10 code lists for cause-specific mortality definitions used in the Article are listed in the appendix (pp 5–11). Finally, we defined years of potential life lost as the difference between age at death and an age threshold of 75 years, in keeping with the definition of premature mortality used elsewhere in this analysis, the constraints of our data with regard to historical death records for older age groups, and the definition of this indicator used in OECD health statistics.

### Statistical analysis

We calculated crude and age-stratified absolute mortality rates for each of the outcomes of interest. We did Poisson regression to obtain hazard ratios (HRs) for each mortality outcome adjusted for age, gender, socioeconomic position (using quintiles of the area-based Scottish Index of Multiple Deprivation measure), and year of follow-up. Due to the presence of an interaction between exposure and year of follow-up, these primary results should be interpreted as the weighted average of the HRs over the 5-year follow-up. To account for this interaction, we did secondary analyses in which HRs were estimated separately for each exposure category and year of follow-up. We also did a secondary analysis to explore the potential differential survival by exposure combination during the exposure period, which might affect results observed during the outcome period (eg, causing bias towards the null if those at highest risk were to die soon after exposure).

To explore whether additional exposures modify the association between a given exposure and all-cause mortality, we calculated stratum-specific HRs and estimates of effect measure modification on both an additive scale (using relative excess risk due to interaction) and a multiplicative scale (using factorial interaction terms within regression models).^18^ We estimated mean years of potential life lost per 100 000 people by exposure combination, based on all-cause mortality before the age of 75 years.

All data cleaning and analysis was done in Stata 15.0, with visualisations created in R version 4.0.3 using *ggplot2*. We used UpSet plots—an alternative to Venn diagrams for more than three sets—to visualise the intersection between the experiences of interest alongside relative hazard for premature mortality.^19^

### Role of the funding source

The funder of the study had no role in study design, data collection, data analysis, data interpretation, or writing of the report**.**

## Results

**Figure 1:**
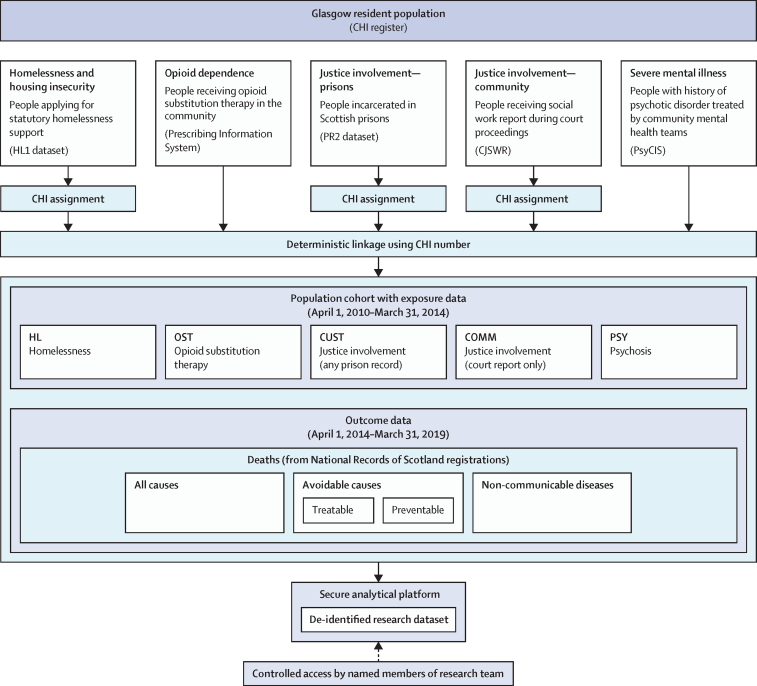
Linkage process for creation of cohort CHI=Community Health Index. CJSWR=Criminal Justice Social Work Report. PsyCIS=Psychosis Clinical Information System.

The cohort for primary analyses consisted of 536 653 adults identified as residents of the Glasgow City Council area who were alive and younger than 75 years at the start of follow-up on April 1, 2014 (figure 1). In brief, for the non-health datasets, 80 083 (91·9%) of 87 142 records could be assigned a CHI number from Criminal Justice Social Work Reports; 60 575 (78·0%) of 77 638 from the HL1 dataset; and 18 984 (76·3%) of 24 887 from the PR2 dataset. These data refer to records rather than individuals and to the entire datasets provided by data controllers, before restriction to the specific dates for this study.

13 075 (2·4%) people made at least one statutory homelessness application during the preceding exposure period; 7412 (1·4%) had at least one episode of opioid substitution therapy dispensing; 5512 (1·0%) were received into prison on at least one occasion; 4619 (0·9%) had at least one court report in the absence of imprisonment; and 3791 (0·7%) were identified in the Psychosis Clinical Information System register (table 2). 28 112 people (5·2%) had any of the experiences of interest; 5178 (1·0%) had more than one.

**Table 2:** Data sources used in cohort creation to ascertain exposures

		**Total (%)**	**Proportion who are male (95% CI)**	**Median age (IQR), years**	Proportion in most deprived SIMD quintile*(95% CI)
Total population		536 653 (100·0%)	52·6% (52·4–52·7)	40·5 (29·5–53·8)	45·6% (45·5–45·7)
No exposures of interest		508 541 (94·8%)	51·9% (51·7–52·0)	40·7 (29·5–54·2)	44·0% (43·9–44·1)
Any exposure of interest		28 112 (5·2%)	64·8% (64·3–65·4)	39·0 (30·5–47·6)	75·2% (74·6–75·7)
Any homelessness		13 075 (2·4%)	54·6% (53·7–55·4)	35·7 (28·7–45·1)	77·8% (77·0–78·5)
	Homelessness only	9463 (1·8%)	46·9% (45·9–48·0)	34·8 (28·1–45·6)	77·4% (76·5–78·2)
	Homelessness and other exposures	3612 (0·7%)	74·5% (73·0–75·9)	37·5 (30·8–44·3)	78·9% (77·5–80·2)
Any opioid dependence		7412 (1·4%)	68·8% (67·7–69·8)	41·7 (36·8–46·5)	80·3% (79·3–81·2)
	Opioid dependence only	4123 (0·8%)	65·3% (63·9–66·8)	42·9 (38·2–47·4)	80·5% (79·2–81·7)
	Opioid dependence and other exposures	3289 (0·6%)	73·1% (71·5–74·6)	40·0 (35·1–45·1)	80·0% (78·5–81·4)
Any custodial justice involvement		5512 (1·0%)	90·9% (90·1–91·6)	35·6 (28·9–44·0)	76·4% (75·1–77·5)
	Custodial justice involvement only	2755 (0·5%)	94·4% (93·4–95·2)	32·7 (27·0–43·0)	74·0% (72·2–75·8)
	Custodial justice involvement and other exposures	2757 (0·5%)	87·4% (86·1–88·6)	37·9 (31·7–44·5)	78·6% (77·0–80·2)
Any community justice involvement		4619 (0·9%)	78·3% (77·0–79·4)	36·4 (28·5–46·4)	73·5% (72·2–74·8)
	Community justice involvement only	3338 (0·6%)	81·7% (80·3–83·0)	35·2 (27·8–46·9)	70·6% (69·0–72·2)
	Community justice involvement and other exposures	1281 (0·2%)	69·4% (66·8–71·9)	38·3 (31·4–45·2)	81·0% (78·7–83·2)
Any psychosis		3791 (0·7%)	57·7% (56·1–59·3)	48·6 (40·0–56·5)	63·4% (61·8–65·0)
	Psychosis only	3255 (0·6%)	55·7% (54·0–57·4)	50·0 (41·5–57·7)	61·2% (59·4–62·9)
	Psychosis and other exposures	536 (0·1%)	70·0% (65·9–73·8)	41·7 (34·9–48·4)	77·2% (73·3–80·7)

Exposure combinations are ordered by frequency of the overall (any) category. SIMD=Scottish Index of Multiple Deprivation.

* Of those with SIMD data available; SIMD data were available for 519 757 (96·8%) of 536 653 in the study cohort. Data for all other demographic variables are complete.

There were 2 502 096 person-years of follow-up, with a mean of 4·7 person-years per individual (SD 1·0). 11 484 individuals died during follow-up (2·1% of cohort), with a further 37 302 individuals (7·0%) leaving the cohort due to migration out of the study area and 21 576 (4·0%) due to turning 75 years of age.

**Figure 2:**
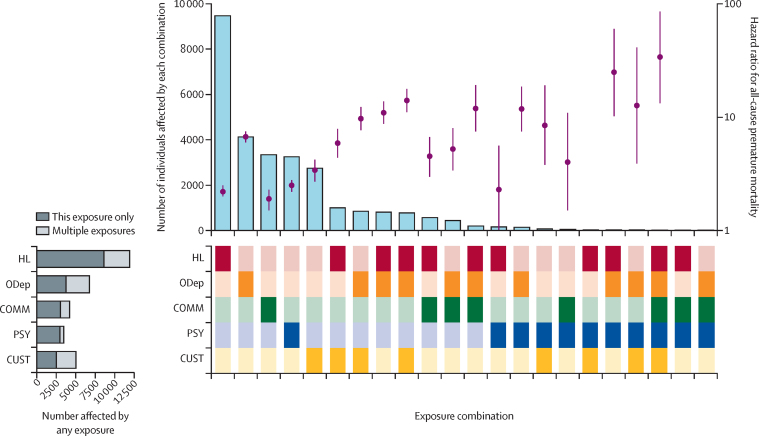
UpSet plot showing frequency of exposure combinations, adjusted HRs with 95% CIs for all-cause premature mortality, and frequency of any exposure Each column corresponds to a specific exposure combination, indicated by the coloured boxes under the X-axis: for example, the left-most (most frequent) exposure combination is homelessness only, whereas the right-most (least frequent) exposure combination is opioid dependence, community justice, and psychosis. For each exposure combination, the vertical bar shows the number of people affected (left-hand Y-axis) and the circle and line show the adjusted HR and 95% CI for premature mortality (right-hand Y-axis). The exposure combinations shown in this graph are ordered by frequency and are mutually exclusive (ie, all individuals in the cohort with any exposure feature in only one category, with no double-counting). The small horizontal bar plot at the bottom left shows the total size of each set—ie, how many individuals had any exposure to that specific experience. This bar is colour-coded according to the number of individuals who had that exposure only (dark shading) versus in combination with other exposures (light shading). HRs are omitted for exposure combinations in which less than three deaths occurred during follow-up: HL, PSY, and COMM; ODep, PSY, and COMM; HL, PSY, and CUST. COMM=community justice involvement. CUST=custodial justice involvement. HL=homelessness and housing insecurity. HR=hazard ratio. ODep=opioid dependence. PSY=psychosis.

All-cause premature mortality rates were substantially higher among people with at least one of the exposures of interest than among those with none, across all age groups (table 3). The additional premature mortality risk conferred by multiple exposures varied by the index exposure: for instance, adjusted HRs were 2·2 (95% CI 1·9–2·5) for homelessness alone versus 8·4 (7·3–9·5) for homelessness in combination with other exposures, compared with 6·7 (6·0–7·5) for opioid dependence alone versus 10·6 (9·4–12·0) for opioid dependence in combination with other exposures (table 3; appendix p 13). Figure 2 illustrates the frequency of each exposure combination in the cohort alongside its associated HR for premature mortality.

**Table 3:** All-cause mortality among the cohort, by exposure status

	**Total number of deaths (person-years at risk)**	**Age-stratified all-cause mortality rate per 100 000 person-years (95% CI)**				Crude HR*(95% CI)	**Adjusted HR**†**(95% CI)**
		18–29 years	30–44 years	45–59 years	60–74 years		
**Exposure status**							
Unexposed	10 103 (2 367 741·8)	12·6 (10·2–15·7)	79·4 (73·3–86·0)	468·8 (452·4–485·9)	1933·0 (1886·2–1981·0)	1·0 (ref)	1·0 (ref)
Any exposure	1381 (134 354·0)	184·1 (142·6–237·6)	801·8 (733·2–876·7)	1752·1 (1620·9–1893·9)	3381·3 (2950·7–3874·8)	2·4 (2·3–2·5)	3·7 (3·5–3·9)
**Homelessness**							
Homelessness only	241 (45 335·5)	45·4 (21·6–95·2)	332·5 (258·7–427·3)	1206·9 (1007·7–1445·6)	3088·1 (2370·9–4022·2)	1·2 (1·1–1·4)	2·2 (1·9–2·5)
Homelessness and other	270 (170 041·3)	488·9 (311·9–766·5)	1529·3 (1298·9–1800·7)	2809·6 (2307·3–3421·3)	3706·9 (1853·8–7412·3)	3·7 (3·3–4·2)	8·4 (7·3–9·5)
**Opioid dependence**							
Opioid dependence only	347 (19 631·2)	615·5 (198·5–1908·4)	1233·4 (1049·3–1449·8)	2598·2 (2246·0–3005·7)	6170·4 (3780·2–10 071·9)	4·1 (3·7–4·6)	6·7 (6·0–7·5)
Opioid dependence and other	310 (15 431·7)	1106·3 (612·7–1997·7)	1636·7 (1410·7–1898·8)	3284·4 (2754·3–3916·5)	3276·8 (461·6–23 262·3)	4·7 (4·2–5·3)	10·6 (9·4–12·0)
**Custodial justice involvement**							
Custodial justice involvement only	88 (13 137·2)	264·5 (156·7–446·7)	493·3 (333·3–730·1)	1481·5 (1068·6–2053·8)	3744·1 (2174·0–6448·1)	1·6 (1·3–1·9)	3·3 (2·6–4·1)
Custodial justice involvement and other	219 (12 948·2)	791·6 (510·7–1226·9)	1501·8 (1248·9–1805·9)	2971·4 (2396·2–3684·6)	2888·0 (931·4–8954·4)	4·0 (3·5–4·5)	9·2 (8·0–10·6)
**Community justice involvement**							
Community justice involvement only	77 (16 302·2)	164·1 (85·4–315·3)	327·1 (211·0–507·0)	791·2 (559·5–1118·9)	2432·4 (1490·2–3970·4)	1·1 (0·9–1·4)	1·8 (1·5–2·3)
Community justice involvement and other	72 (6154·0)	296·9 (111·4–791·2)	1069·7 (768·0–1489·8)	1877·4 (1287·5–2737·6)	6207·9 (2788·9–13 818·0)	2·7 (2·2–3·5)	5·5 (4·3–7·0)
**Psychosis**							
Psychosis only	227 (15 491·5)	94·1 (13·3–667·9)	551·6 (369·7–822·9)	1449·2 (1196·9–1754·7)	3425·2 (2807·1–4179·4)	3·4 (3·0–3·9)	2·5 (2·1–2·8)
Psychosis and other	49 (2533·4)	921·6 (297·2–2857·5)	1579·6 (1019·1–2448·4)	2698·5 (1808·7–4026·0)	3818·6 (955·0–15 268·5)	4·5 (3·4–6·0)	7·3 (5·5–9·8)

Exposure combinations are ordered by frequency of the overall (any) category. HR=hazard ratio. SIMD=Scottish Index of Multiple Deprivation.

* Unexposed population as reference group.

† Unexposed population as reference group; adjusted for age, gender, SIMD quintile, and calendar time.

Secondary analyses examining mortality during the exposure period suggested that the overall pattern was similar to that observed during the outcome period, although effect estimates were higher during the outcome period than during the exposure period (appendix pp 21–22). Secondary analyses incorporating an interaction between exposure and year of follow-up yielded broadly similar results, although the effect estimate for multiple exposures tended to vary somewhat over the period of follow-up (appendix pp 14–20).

For most exposures, additional exposures acted as positive effect modifiers of all-cause mortality on the additive scale (ie, that the presence of additional exposures was associated with an increased hazard over and above that expected from their sum; appendix p 23). There was little evidence of effect modification by additional exposures on the multiplicative scale, except for opioid dependence, for which this effect was negative (appendix p 23).

**Figure 3:**
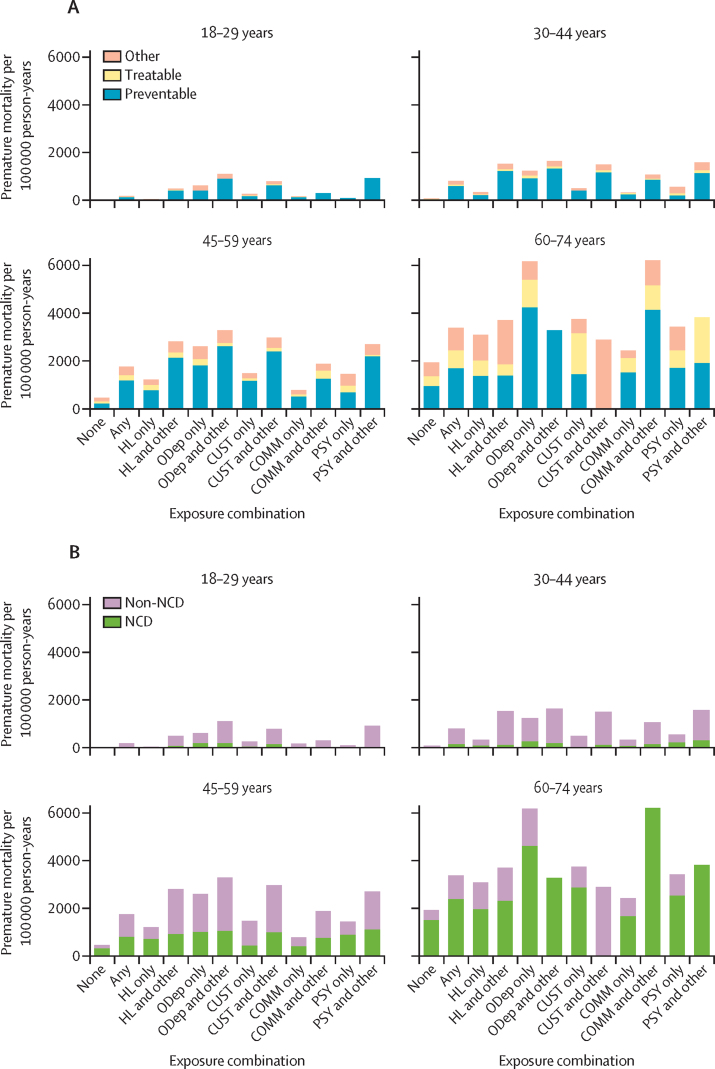
Age-stratified mortality rates per 100 000 person-years, by exposure combination and cause Premature mortality due to avoidable causes, comprising preventable and treatable causes (A), and non-communicable diseases, comprising cancer, cardiovascular disease, chronic respiratory disease, and diabetes (B). Exposure combinations are ordered by frequency of any flag for that exposure. COMM=community justice involvement. CUST=custodial justice involvement. HL=homelessness and housing insecurity. NCD=non-communicable disease. ODep=opioid dependence. PSY=psychosis.

The proportion, absolute rate, and HR of death from causes deemed avoidable were consistently higher in people with any versus no exposures of interest, and in people with multiple rather than single exposures for almost all age groups (figure 3A; appendix pp 24–25). Most deaths from avoidable causes among exposed individuals were accounted for by preventable deaths, with treatable deaths making up a smaller fraction; this finding was more pronounced among those with multiple exposures.

The proportion of deaths attributed to non-communicable diseases (ie, cancer, cardiovascular disease, diabetes, and chronic respiratory disease) was lower in people with the exposures of interest than in those unexposed, and in people with multiple versus single exposures (appendix pp 26–27). However, absolute rates and HRs for deaths from non-communicable diseases were higher in those with any exposure, and for most instances of multiple versus single exposures (figure 3B; appendix pp 26–27).

The mean years of potential life lost per decedent was higher for all exposure combinations than for the unexposed group, ranging from 15·3 (95% CI 14·1–16·5) for psychosis only to 33·9 (30·6–37·1) for the combination of homelessness and housing insecurity, opioid dependence, and community justice involvement (appendix pp 28–30). Mean years of potential life lost per 100 000 people at risk, which provides an indication of population burden, was also substantially higher for all exposure combinations than for the unexposed group, with the highest burden associated with combinations involving opioid dependence (appendix pp 28–30).

## Discussion

Using cross-sectoral administrative data linkage from local authority, health-care, justice, and vital registration systems, we found that people with co-occurring experiences of homelessness, justice involvement, opioid dependence, and psychosis had high rates of premature mortality compared with individuals with one or none of these experiences. However, the impact of multiple disadvantage varied; for instance, people with opioid dependence had high premature mortality regardless of whether they had co-occurring experiences. The rate and proportion of deaths from avoidable causes among people with multiple disadvantages was higher than among people with only one for almost all age groups, which was in turn higher than among those with none; most of these avoidable deaths were accounted for by conditions preventable through public health and primary prevention. Although non-communicable disease accounted for a lower proportion of deaths among people with one or multiple disadvantages than among people with none, absolute rates and relative hazards of mortality from non-communicable disease were higher for any versus no exposures of interest, and for most combinations of multiple versus single exposures.

Our finding that multiple disadvantage was generally associated with increased mortality, but that this association varies by the individual exposures involved, is consistent with existing literature,^5^, ^9^ and might facilitate the identification and support of subgroups at particularly high risk of poor outcomes. However, it is notable that mortality was substantially increased even among those only exposed to one form of disadvantage. For instance, individuals with homelessness alone accounted for almost 2% of the Glasgow population yet had a 2·4-times greater hazard of premature death than their unexposed peers. Similarly, among people with a history of imprisonment, HRs for premature mortality were 3·4-times greater than those for the unaffected population, even in the absence of other well established risk factors such as opioid dependence or homelessness.^9^, ^20^, ^21^ These findings suggest the need for wide-ranging policy and service efforts across the population to prevent these experiences and mitigate associated poor health outcomes.

The high burden of avoidable mortality associated with the intersection between these experiences is notable as, to our knowledge, only one previous study has investigated this question, and this study only examined the combination of severe mental illness and substance use.^22^ Our results extend previous research showing that each of these experiences in isolation is associated with a substantial increase in the risk of death from avoidable causes.^23^, ^24^, ^25^, ^26^ Together, these findings suggest that current public health and health-care provision is failing to benefit many of those with the experiences of interest, creating unjust inequalities in risk of death.

Our findings also contribute to an underdeveloped evidence base on the burden of non-communicable diseases among people affected by single and multiple forms of disadvantage.^5^, ^9^ Other studies have found that access to prevention and treatment for common physical health conditions among people experiencing social marginalisation and exclusion is often poor.^27^, ^28^, ^29^ Current priorities for service delivery and research activity with these populations tend to be dominated by the prevention and management of infections and external causes: our findings suggest that this does not adequately reflect their true burden of ill health, to which non-communicable diseases make a substantial and probably increasing contribution, and that greater attention must be paid to the prevention and treatment of common long-term conditions.

Strengths of this study include its population-based approach, which enables us to assess the associations between diverse exposure combinations and mortality in comparison with an unexposed population, rather than assessing risk factors for mortality among people selected on the basis of an index exposure. Although the exposures under investigation in this study do not represent an exhaustive set of identities or experiences associated with social exclusion, their occurrence and outcomes are heavily influenced by policy choices (eg, relating to the housing market, poverty and social security, or justice and sentencing policy), and they are tractable to study through existing datasets collected routinely in Scotland, making them an ideal focus for social epidemiology.

The use of linked administrative and registry data maximises coverage and ascertainment, and reduces the risk of threats to validity from participation and attrition biases, which are common in traditional cohort studies with people experiencing social disadvantage and exclusion.

In this study, complete ascertainment of the exposure of interest was only possible for imprisonment (through the use of national prison records); other datasets will have varying degrees of under-ascertainment, as recording depends on service access, uptake, and eligibility. The direction of bias this under-ascertainment might cause is unclear, as severity of disadvantage might either increase or decrease the likelihood of ascertainment, depending on individual and service factors. However, our use of 4 years of exposure data maximises the chances of inclusion even in situations in which engagement with services is sporadic or short-lived, and a previous analysis indicated that changes in the length of the study period had a small effect on prevalence estimates for most exposures (Tweed, unpublished). Other sources suggest that ascertainment from these datasets is likely to be fairly high: for instance, a national survey of people accessing injecting equipment providers in Scotland during the study period found that between 88% and 90% had received prescribed methadone at any timepoint and 71–76% had done so in the past 6 months,^30^ whereas data from the Scottish Household Survey around the same time found that 60–65% of people reporting a history of homelessness had approached their local authority for help during the most recent episode (although this survey is potentially biased by being restricted to those individuals now living in private households). Ascertainment might be strengthened in future work through triangulation with additional administrative datasets (eg, from third sector as well as statutory services) or data from primary research.

However, a further consideration is that the extent to which records from non-health sources (including the PR2 dataset) could be assigned a CHI number—and therefore included in the linked cohort—varied between datasets. Failure to identify a CHI number might be explained by migration out of the NHS Greater Glasgow and Clyde area following exposure (as the CHI register is a live database, in contrast to the retrospective exposure datasets) or incorrect identifiers in one or multiple datasets, but also reflects the use of a fairly stringent matching algorithm likely to prioritise specificity over sensitivity. The CHI register is updated automatically when individuals register with health services in other areas and is subject to regular checks on residence, although there might still be some degree of under-ascertainment of migration (eg, due to individual delays in re-registration). Therefore, exclusion of records for which a CHI could not be assigned is an important limitation that could be addressed in future work using national (rather than regional) population registers, prospective rather than retrospective linkages, and threshold-based approaches to probabilistic linkage permitting sensitivity analyses. Developments in data access, to enable use of national-level data for exposures and outcomes, would also enhance the generalisability of our findings.

The use of avoidable mortality among people with these experiences is novel and offers new insights into opportunities for services to intervene. Our findings would be enhanced by further work to disentangle the relative contribution of incidence and case-fatality to the observed burden of avoidable mortality. The definition of causes as avoidable is subject to debate, and to change over time as knowledge and technologies develop;^14^ moreover, many instances of these experiences are themselves avoidable through wider social policy measures in the realms of welfare, employment, housing, and justice.^31^, ^32^

Limitations in data availability meant that we classified exposure using a cumulative approach across a 4-year period, and treated exposure and follow-up periods separately. Improvements to data access in the future should enable exposures to be assessed and modelled on a time-varying basis, and the impact of event timing explored. Similar constraints meant we were unable to account for periods of incarceration during follow-up, during which mortality risk might differ.^33^ However, previous modelling suggests the overall impact of this is likely to be small^21^ and, in our study, the median in-prison time during the exposure period among those imprisoned was only 9% (equivalent to 130 of 1460 days [IQR 44–334]; Tweed, unpublished). We did not have data on heterogeneity within our exposure categories—for instance, polysubstance use or treatment access among people with opioid dependence—which might have affected mortality risk.

Nonetheless, our findings show the value of administrative data linkage in understanding the health experiences of people for whom participation in primary research can be challenging. Future work should extend these methods to better understand the causal pathways underlying excess mortality, for instance by examining the timing and sequencing of exposures; investigating effect modification by factors such as gender and socioeconomic position; and evaluating natural experiments that affect exposure to the experiences of interest.

At present, such cross-sectoral linkage is often resource-intensive and time-consuming, hindering efforts to monitor trends and evaluate interventions at the population level, and to support joined-up care provision and multi-agency working at the individual level. Therefore, investment in routine and responsive linkage across multiple sectors might facilitate service and policy responses that are not only better informed by evidence but also more holistic in their approach.

## Data sharing

The datasets used in the study can be requested for use in other research studies, subject to obtaining necessary permissions from the relevant data controllers, by contacting the West of Scotland Safe Haven (https://www.nhsggc.org.uk/about-us/professional-support-sites/glasgow-safe-haven/).

## Declaration of interests

We declare no competing interests.

## References

[1] Shinn M (2010). Homelessness, poverty, and social exclusion in the United States and Europe. Eur J Homelessness.

[2] March JC, Oviedo-Joekes E, Romero M (2006). Drugs and social exclusion in ten European cities. Eur Addict Res.

[3] Allman D (2013). The sociology of social inclusion. SAGE Open.

[4] Smith D, Stewart J (1997). Probation and social exclusion. Soc Policy Adm.

[5] Aldridge RW, Story A, Hwang SW (2018). Morbidity and mortality in homeless individuals, prisoners, sex workers, and individuals with substance use disorders in high-income countries: a systematic review and meta-analysis. Lancet.

[6] Somers JM, Moniruzzaman A, Rezansoff SN, Brink J, Russolillo A (2016). The prevalence and geographic distribution of complex co-occurring disorders: a population study. Epidemiol Psychiatr Sci.

[7] Bramley G, Fitzpatrick S, Wood J (2019).

[8] Bramley G, Fitzpatrick S, Sosenko F (2020). Mapping the “hard edges” of disadvantage in England: adults involved in homelessness, substance misuse, and offending. Geogr J.

[9] Tweed EJ, Thomson RM, Lewer D (2021). Health of people experiencing co-occurring homelessness, imprisonment, substance use, sex work and/or severe mental illness in high-income countries: a systematic review and meta-analysis. J Epidemiol Community Health.

[10] National Institute for Health and Care Excellence (2017).

[11] National Institute for Health and Care Excellence (2016).

[12] Jutte DP, Roos LL, Brownell MD (2011). Administrative record linkage as a tool for public health research. Annu Rev Public Health.

[13] Lyons RA, Ford DV, Moore L, Rodgers SE (2014). Use of data linkage to measure the population health effect of non-health-care interventions. Lancet.

[14] Castelli A, Nizalova A (2011).

[15] Nolte E, McKee M (March 1, 2004). Does health care save lives? Avoidable mortality revisited. https://www.nuffieldtrust.org.uk/research/does-healthcare-save-lives-avoidable-mortality-revisited.

[16] Organisation for Economic Co-operation and Development, Eurostat (November, 2019). Avoidable mortality: OECD/Eurostat lists of preventable and treatable causes of death. https://www.oecd.org/health/health-systems/Avoidable-mortality-2019-Joint-OECD-Eurostat-List-preventable-treatable-causes-of-death.pdf.

[17] WHO (2014).

[18] Knol MJ, VanderWeele TJ (2012). Recommendations for presenting analyses of effect modification and interaction. Int J Epidemiol.

[19] Lex A, Gehlenborg N (2014). Sets and intersections. Nat Methods.

[20] Chang Z, Lichtenstein P, Larsson H, Fazel S (2015). Substance use disorders, psychiatric disorders, and mortality after release from prison: a nationwide longitudinal cohort study. Lancet Psychiatry.

[21] Kinner SA, Forsyth S, Williams G (2013). Systematic review of record linkage studies of mortality in ex-prisoners: why (good) methods matter. Addiction.

[22] Lumme S, Pirkola S, Manderbacka K, Keskimäki I (2016). Excess mortality in patients with severe mental disorders in 1996–2010 in Finland. PLoS One.

[23] Hoang U, Goldacre MJ, Stewart R (2013). Avoidable mortality in people with schizophrenia or bipolar disorder in England. Acta Psychiatr Scand.

[24] Degenhardt L, Larney S, Randall D, Burns L, Hall W (2014). Causes of death in a cohort treated for opioid dependence between 1985 and 2005. Addiction.

[25] Onyeka IN, Beynon CM, Vohlonen I (2015). Potential years of life lost due to premature mortality among treatment-seeking illicit drug users in Finland. J Community Health.

[26] Aldridge RW, Menezes D, Lewer D (2019). Causes of death among homeless people: a population-based cross-sectional study of linked hospitalisation and mortality data in England. Wellcome Open Res.

[27] Fraser J, Maycock M, Meek R, Woodall J (2021). Issues and innovations in prison health research: methods, issues and innovations.

[28] Liu M, Hwang SW (2021). Health care for homeless people. Nat Rev Dis Primers.

[29] Mitchell AJ, Malone D, Doebbeling CC (2009). Quality of medical care for people with and without comorbid mental illness and substance misuse: systematic review of comparative studies. Br J Psychiatry.

[30] Health Protection Scotland, Glasgow Caledonian University, West of Scotland Specialist Virology Centre (2019).

[31] Goldblatt P, Lewis C (1998).

[32] Advisory Council on the Misuse of Drugs (2015).

[33] Graham L, Fischbacher CM, Stockton D, Fraser A, Fleming M, Greig K (2015). Understanding extreme mortality among prisoners: a national cohort study in Scotland using data linkage. Eur J Public Health.

