# Supplementary material for: Premature mortality in people affected by co-occurring homelessness, justice involvement, opioid dependence, and psychosis: a retrospective cohort study using linked administrative data
Source: Lancet Public Health. 2022 Jul 28;7(9):e733–43. doi: 10.1016/S2468-2667(22)00159-1 (PMC9433331; doi:10.1016/S2468-2667(22)00159-1)
Supplement: Supplementary appendix [file mmc1.pdf]

THE LANCET
Public Health

Supplementary appendix

This appendix formed part of the original submission and has been peer reviewed. We post it as supplied by the authors.

Supplement to: Tweed EJ, Leyland AH, Morrison D, Katikireddi SV. Premature mortality in people affected by co-occurring homelessness, justice involvement, opioid dependence, and psychosis: a retrospective cohort study using linked administrative data. *Lancet Public Health* 2022; published online July 27. https://doi.org/10.1016/S2468-2667(22)00159-1.

SUPPLEMENTARY MATERIAL

Section S1. LINKAGE PROCESS

CHI seeding – the process of matching records from administrative datasets not containing a Community Health Index (CHI) number to the CHI register – was undertaken by the West of Scotland Safe Haven for the HL1, PR2, and CJSWR datasets. The CJSWR dataset already contained CHI numbers for some records, reflecting the incorporation of the criminal justice social work function into Glasgow City Health and Social Care Partnership as part of health and social care integration during the study period.

Before seeding, datasets were checked for consistency in the formatting and length of fields.

CHI seeding was carried out on a deterministic basis supplemented by manual review, using forename, surname, and date of birth OR forename & surname soundex codes combined with date of birth. Soundex codes are anonymised representation of surnames consisting of the initial letter of the surname and three digits, used to enable matching of names despite spelling variations (e.g., Mohammed vs Muhamed, MacDonald vs McDonald). Manual assessments of postcodes were included in supplementary reviews but not in the primary matching process due to migration over time potentially resulting in false negatives.

No reference datasets with known true- and false-matches were available to assess sensitivity or specificity of the CHI seeding process.

Once CHI seeding was complete, all linkages were undertaken on a deterministic basis using CHI numbers.

Results of linkage

The flow charts below describe the linkage process for each of the non-health datasets requiring CHI seeding. CHI seeding was undertaken on the entire datasets provided by the data controllers, which were not restricted to the specific dates of this study: totals for the number of records and of unique individuals may therefore not match those presented in the results for this article.

In contrast to the HL1 and CJSWR datasets, which originated from Glasgow City Health and Social Care Partnership and whose geographical scope therefore fell entirely within the area covered by the West of Scotland Safe Haven, the PR2 (prisons) dataset was national. This allowed us to identify Glasgow City residents who had experienced imprisonment regardless of where in Scotland they had been imprisoned.

Figure S1.1. Flowchart demonstrating CHI seeding process for HL1 dataset

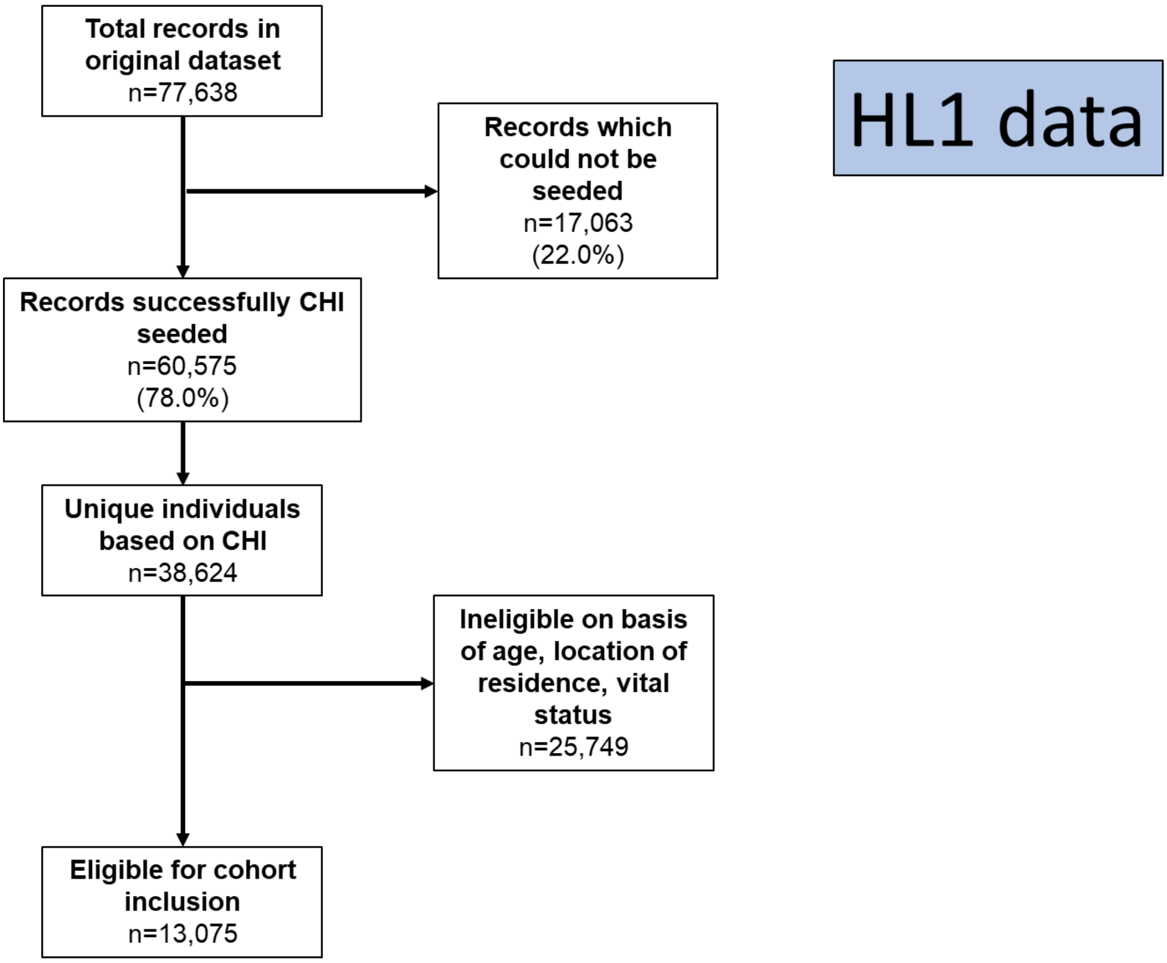

Table S1.1. Comparison of demographic characteristics between records in HL1 dataset which could and could not be assigned a CHI number (Note that the figures below relate to records, rather than individuals)

HL1	Able to be assigned a CHI		
	**Yes**	**No**	**Total**
**Total number of records (%)**	60,575 (78.0)	17,063 (22.0)	77,638 (100.0)
**Percentage male**	57.9	52.5	57.9
**Mean age**	44	43	44

Figure S1.2. Flowchart demonstrating CHI seeding process for CJSWR dataset

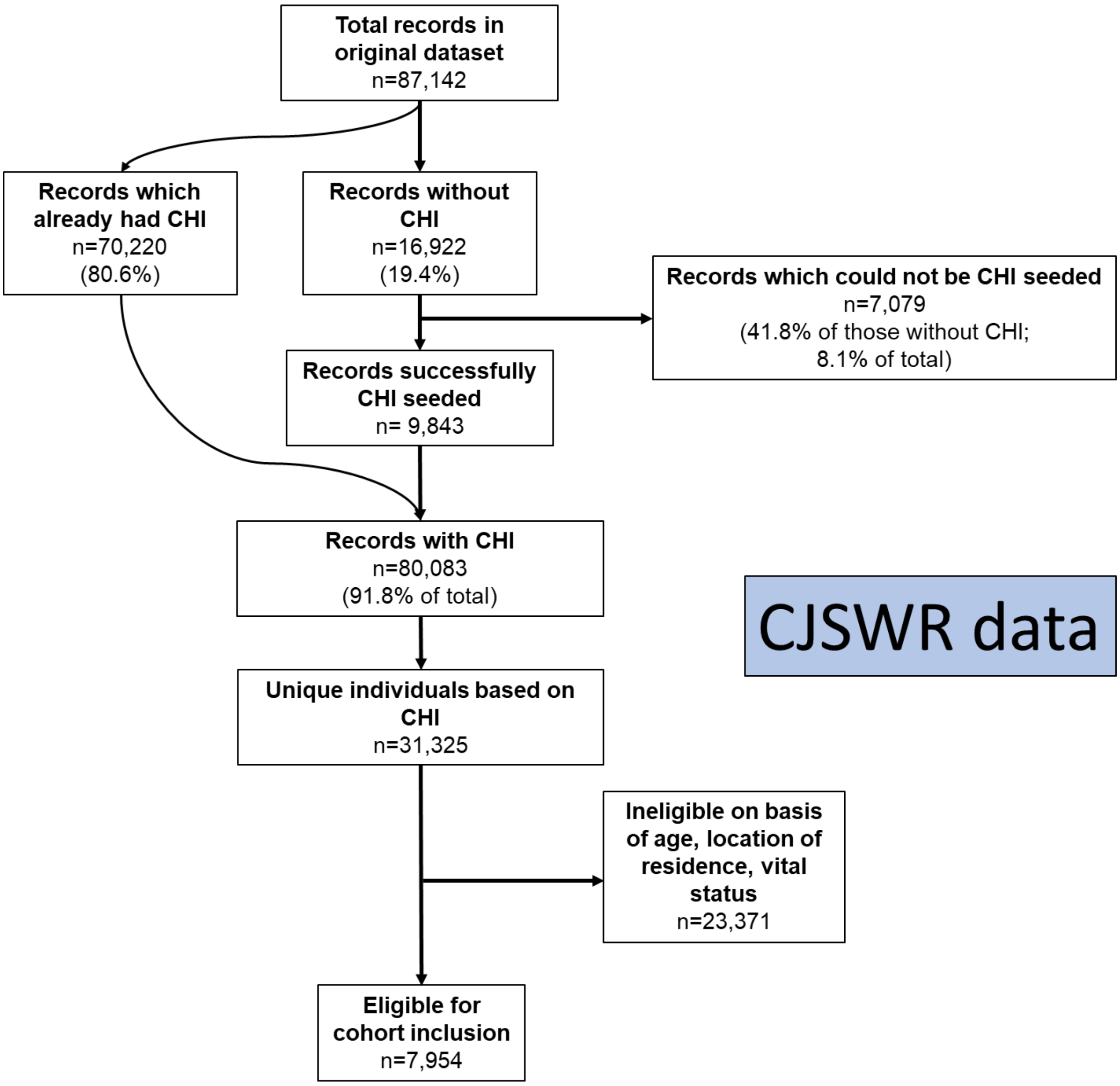

Table S1.2. Comparison of demographic characteristics between records in CJSWR dataset which could and could not be assigned a CHI number. (Note that the figures below relate to records, rather than individuals; records for whom a CHI number was available in the original CJSWR dataset are included under the 'Yes' category)

CJSWR	Able to be assigned a CHI		
	Yes	No	Total
Total number of records (%)	80,083 (91.8)	7,079 (8.2)	87,142 (100.0)
Percentage male	85.4	85.0	85.4
Mean age	42	44	42

Figure S1.3. Flowchart demonstrating CHI seeding process for PR2 dataset

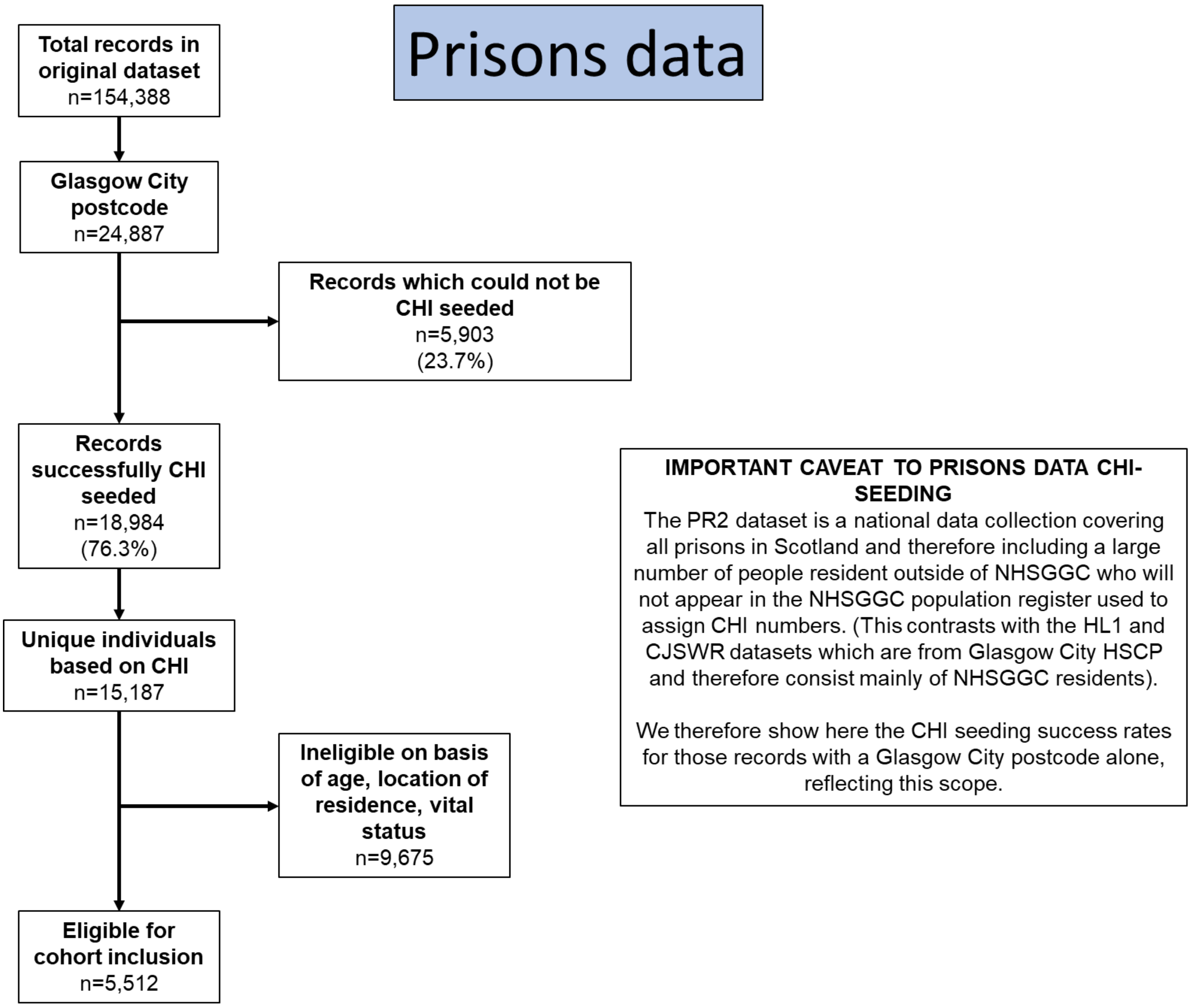

Table S1.3. Comparison of demographic characteristics between records in PR2 dataset which could and could not be assigned a CHI number. (Note that the figures below relate to records, rather than individuals, and to the overall national dataset from all prisons comprising 154,388 records. Many of those who could not be assigned a CHI number were therefore people resident outside of NHSGGC who will not be included in the NHSGGC population register used to identify CHI numbers.)

PR2	Able to be assigned a CHI		
	Yes	No	Total
**Total number of records (%)**	42,541 (27.6)	100,926 (72.4)	154,388 (100.0)
**Percentage male**	92.1	90.2	90.7
**Mean age**	43	42	42

Section S2. List of ICD-10 codes used in classification of mortality from avoidable causes of death, comprising preventable and treatable causes

Source:

OECD/Eurostat (2019). Avoidable mortality: OECD/Eurostat lists of preventable and treatable causes of death (November 2019 version). https://www.oecd.org/health/health-systems/Avoidable-mortality-2019-Joint-OECD-Eurostat-List-preventable-treatable-causes-of-death.pdf

All of the causes listed below have an age threshold of 0-74 years of age for inclusion in the definition of avoidable mortality.

Causes of death	Preventable	Treatable	ICD-10 code(s)	Stated rationale (from OECD/Eurostat guidance)
Intestinal diseases	X		A00-A09	Most of these infections can be prevented through prevention measures (e.g. improve water and food safety)
Diphtheria, Tetanus, Poliomyelitis	X		A35, A36, A80	Most of these infections can be prevented through vaccination
Whooping cough	X		A37	Most of these infections can be prevented through vaccination
Meningococcal infection	X		A39	Most of these infections can be prevented through vaccination
Sepsis due to streptococcus pneumonia and sepsis due to Haemophilus influenzae	X		A40.3, , A41.3	Most of these infections can be prevented through vaccination
Haemophilus influenzae infections	X		A49.2	Most of these infections can be prevented through vaccination
Sexually transmitted infections (except HIV/AIDS)	X		A50-A60, A63, A64	These infections can be prevented through prevention measures.
Varicella	X		B01	Most of these infections can be prevented through vaccination.
Measles	X		B05	Most of these infections can be prevented through vaccination.
Rubella	X		B06	Most of these infections can be prevented through vaccination.
Viral hepatitis	X		B15-B19	This condition is preventable and will not require treatment if prevented
HIV/AIDS	X		B20-B24	This condition is preventable and will not require treatment if prevented
Malaria	X		B50-B54	This condition is preventable and will not require treatment if prevented
Haemophilus and pneumococcal meningitis	X		G00.0, G00.1	Most of these infections can be prevented through vaccination.
Tuberculosis	X (50%)	X (50%)	A15-A19, B90, J65	Reduction in deaths from tuberculosis in several countries has been about evenly achieved through greater prevention (reduction in incidence) and earlier detection and more effective treatment (higher survival rates).

Causes of death	Preventable	Treatable	ICD-10 code(s)	Stated rationale (from OECD/Eurostat guidance)
Scarlet fever		X	A38	Case-fatality rates can be reduced through early detection and appropriate antibiotic treatment
Sepsis		X	A40 (excl. A40.3),A41 (excl. A41.3)	Case-fatality rates can be reduced through greater quality of care and reduced patient adverse events, and early detection and appropriate antibiotic treatment
Cellulitis		X	A46, L03	Case-fatality rates can be reduced through early detection and appropriate antibiotic treatment.
Legionnaires disease		X	A48.1	Case-fatality rates can be reduced through early detection and appropriate antibiotic treatment.
Streptococcal and enterococci infection		X	A49.1	Case-fatality rates can be reduced through early detection and appropriate antibiotic treatment.
Other meningitis		X	G00.2, G00.3, G00.8, G00.9	Case-fatality rates can be reduced through early detection and appropriate antibiotic treatment.
Meningitis due to other and unspecified causes		X	G03	Case-fatality rates can be reduced through early detection and appropriate antibiotic treatment.
Lip, oral cavity and pharynx cancer	X		C00-C14	This condition can be largely prevented through prevention measures (e.g. reduce smoking).
Oesophageal cancer	X		C15	This condition can be largely prevented through prevention measures (e.g. reduce smoking).
Stomach cancer	X		C16	This condition can be largely prevented through prevention measures (e.g. reduce smoking and alcohol consumption, and improve nutrition).
Liver cancer	X		C22	This condition can be largely prevented through prevention measures (e.g. reduce smoking and alcohol consumption).
Lung cancer	X		C33-C34	This condition can be largely prevented through prevention measures (e.g., reduce smoking).
Mesothelioma	X		C45	This condition can be largely prevented through prevention measures (e.g. reduce asbestos exposure).
Skin (melanoma) cancer	X		C43	This condition can be largely prevented through prevention measures (e.g. reduce sun exposure).
Bladder cancer	X		C67	This condition can be largely prevented through prevention measures (e.g., reduce smoking).
Cervical cancer	X (50%)	X (50%)	C53	Cervical cancer can be prevented through vaccination and screening can also find pre-cancerous abnormalities that can be treated to prevent cancer, but five-year survival after cancer detection is also relatively high and rising.
Colorectal cancer		X	C18-C21	Case-fatality rates have been reduced through earlier detection and treatment. Five-year survival after detection is relatively high and rising.
Breast cancer (female only)		X	C50	Case-fatality rates have been reduced through earlier detection and treatment. Five-year survival after detection is relatively high and rising.

Causes of death	Preventable	Treatable	ICD-10 code(s)	Stated rationale (from OECD/Eurostat guidance)
Uterus cancer		X	C54, C55	Case-fatality rates have been reduced through earlier detection and treatment. Five-year survival after detection is relatively high and rising.
Testicular cancer		X	C62	Case-fatality rates have been reduced through earlier detection and treatment. Five-year survival after detection is relatively high and rising.
Thyroid cancer		X	C73	Case-fatality rates have been reduced through early detection and appropriate treatment.
Hodgkin's disease		X	C81	Case-fatality rates have been reduced through early detection and appropriate treatment.
Lymphoid leukaemia		X	C91.0, C91.1	Case-fatality rates have been reduced through early detection and appropriate treatment.
Benign neoplasm		X	D10-D36	Case-fatality rates have been reduced through early detection and appropriate treatment.
Nutritional deficiency anaemia	X		D50-D53	This condition can be largely prevented through prevention measures (e.g. improve nutrition).
Diabetes mellitus	X (50%)	X (50%)	E10-E14	Type 1 diabetes is not preventable, but appropriate treatments can reduce mortality. Type 2 diabetes is largely preventable (e.g. improve nutrition), but appropriate treatments can also reduce mortality.
Thyroid disorders		X	E00-E07	Case-fatality rates can be reduced through early detection and appropriate treatment.
Adrenal disorders		X	E24-E25 (except E24.4), E27	Case-fatality rates can be reduced through early detection and appropriate treatment.
Epilepsy		X		
Aortic aneurysm	X (50%)	X (50%)	I71	This condition is both preventable through prevention measures (similar risk factors as for ischaemic heart diseases) and treatable.
Hypertensive diseases	X (50%)	X (50%)	I10-I13, I15	This condition is both preventable through prevention measures (e.g. reduce smoking, improve nutrition and physical activity) and treatable.
Ischaemic heart diseases	X (50%)	X (50%)	I20-I25	Reduction in deaths from IHD over the past decades in several countries has been about evenly achieved through greater prevention (reduction in incidence) and earlier detection and more effective treatment (higher survival rates).
Cerebrovascular diseases	X (50%)	X (50%)	I60-I69	Reduction in deaths from CVD over the past decades in several countries has been about evenly achieved through greater prevention (reduction in incidence) and earlier detection and more effective treatment (higher survival rates).
Other atherosclerosis	X (50%)	X (50%)	I70, I73.9	This condition is both preventable through prevention measures (e.g. improve nutrition) and treatable.
Rheumatic and other heart disease		X	I00-I09	Case-fatality rates can be reduced through appropriate treatment.

Causes of death	Preventable	Treatable	ICD-10 code(s)	Stated rationale (from OECD/Eurostat guidance)
Venous thromboembolism		X*	I26, I80, I82.9	The majority of venous thrombosis events result from hospitalisations. These cases are treatable to the extent that they are linked to the quality of care that people receive.
Influenza		X	J09-J11	Most of the deaths can be prevented through prevention measures (e.g. vaccination).
Pneumonia due to Streptococcus pneumonia or Haemophilus influenzae	X		J13-J14	Most of these infections can be prevented through vaccination.
Chronic lower respiratory diseases	X		J40-J44	This condition can be largely prevented through prevention measures (e.g. reduce smoking).
Lung diseases due to external agents	X		J60-J64, J66-J70, J82, J92	This condition can be largely prevented through prevention measures (e.g. reduce exposure to chemical, gases and other agents).
Upper respiratory infections		X	J00-J06, J30-J39	Case-fatality rates can be reduced through appropriate treatment.
Pneumonia, not elsewhere classified or organism unspecified		X	J12, J15, J16- J18	Case-fatality rates can be reduced through early detection and appropriate antibiotic treatment.
Acute lower respiratory infections		X	J20-J22	Case-fatality rates can be reduced through appropriate treatment.
Asthma and bronchiectasis		X	J45-J47	Case-fatality rates can be reduced through appropriate treatment (e.g. medication).
Adult respiratory distress syndrome		X	J80	Case-fatality rates can be reduced through appropriate treatment.
Pulmonary oedema		X	J81	Case-fatality rates can be reduced through appropriate treatment.
Abscess of lung and mediastinum pyothorax		X	J85, J86	Case-fatality rates can be reduced through appropriate treatment.
Other pleural disorders		X	J90, J93, J94	Case-fatality rates can be reduced through appropriate treatment.
Gastric and duodenal ulcer		X	K25-K28	Case-fatality rates can be reduced through early detection and appropriate treatment.
Appendicitis		X	K35-K38	Case-fatality rates can be reduced through early detection and appropriate treatment.
Abdominal hernia		X	K40-K46	Case-fatality rates can be reduced through early detection and appropriate treatment.
Cholelithiasis and cholecystitis		X	K80-K81	Case-fatality rates can be reduced through early detection and appropriate treatment.
Other diseases of gallbladder or biliary tract		X	K82-K83	Case-fatality rates can be reduced through early detection and appropriate treatment.
Acute pancreatitis		X	K85.0,1,3,8,9	Case-fatality rates can be reduced through early detection and appropriate treatment.
Other diseases of pancreas		X	K86.1,2,3,8,9	Case-fatality rates can be reduced through early detection and appropriate treatment.

Causes of death	Preventable	Treatable	ICD-10 code(s)	Stated rationale (from OECD/Eurostat guidance)
Nephritis and nephrosis		X	N00-N07	Case-fatality rates can be reduced through early detection and appropriate treatment.
Obstructive uropathy		X	N13,N20-N21, N35	Case-fatality rates can be reduced through early detection and appropriate treatment.
Renal failure		X	N17-N19	Case-fatality rates can be reduced through early detection and appropriate treatment.
Renal colic		X	N23	Case-fatality rates can be reduced through early detection and appropriate treatment.
Disorders resulting from renal tubular dysfunction		X	N25	Case-fatality rates can be reduced through early detection and appropriate treatment.
Unspecified contracted kidney, small kidney of unknown cause		X	N26-N27	Case-fatality rates can be reduced through early detection and appropriate treatment.
Inflammatory diseases of genitourinary system		X	N34.1,N70-N73,N75.0,N75.1,N76.4,6	Case-fatality rates can be reduced through early detection and appropriate treatment.
Prostatic hyperplasia		X	N40	Case-fatality rates can be reduced through early detection and appropriate treatment.
Tetanus neonatorum	X		A33	Most of these infections can be prevented through vaccination.
Obstetrical tetanus	X		A34	Most of these infections can be prevented through vaccination.
Pregnancy, childbirth and the puerperium		X	O00-O99	Effective treatment is available in most cases to avoid maternal mortality.
Certain conditions originating in the perinatal period		X	P00-P96	Case-fatality rates can be reduced through early detection and appropriate treatment.
Certain congenital malformations (neural tube defects)	X		Q00, Q01, Q05	These conditions can be prevented through prevention measures (improve maternal nutrition, e.g. folic acid consumption).
Congenital malformations of the circulatory system (heart defects)		X	Q20-Q28	These conditions can be treated through surgical operations
Drugs, medicaments and biological substances causing adverse effects in therapeutic use		X*	Y40-Y59	These conditions are treatable through better drug prescription and adherence.
Misadventures to patients during surgical and medical care		X*	Y60-Y69,Y83-Y84	These conditions are treatable through better quality of care that patients receive.
Medical devices associated with		X*	Y70–Y82	These conditions are treatable through better quality of care that patients receive.

Causes of death	Preventable	Treatable	ICD-10 code(s)	Stated rationale (from OECD/Eurostat guidance)
adverse incidents in diagnostic and therapeutic use				
Transport Accidents	X		V01-V99	Deaths can be prevented through public health interventions (e.g. road safety measures).
Accidental Injuries	X		W00-X39, X46-X59	Deaths can be prevented through public health interventions (e.g. injury prevention campaigns).
Intentional self-harm	X		X66-X84	Deaths can be prevented through public health interventions (e.g. suicide prevention campaigns).
Event of undetermined intent	X		Y16-Y34	Deaths can be prevented through public health interventions (e.g. harm prevention campaigns).
Assault	X		X86-Y09	Deaths can be prevented through public health interventions.
Alcohol-specific disorders and poisonings	X		E24.4, F10, G31.2, G62.1, G72.1, I42.6, K29.2, K70, K85.2, K86.0, Q86.0, R78.0, X45, X65, Y15	Deaths can be largely prevented through public health interventions (e.g. alcohol control policies).
Other alcohol-related disorders	X		K73, K74.0-K74.2, K74.6	Deaths can be largely prevented through public health interventions (e.g. alcohol control policies).
Drug disorders and poisonings**	X		F11-F16, F18-F19, X40-X44, X85, Y10-Y14	Deaths can be largely prevented through public health interventions (e.g. drug control policies).
Intentional self-poisoning by drugs**	X		X60-X64	Deaths can be largely prevented through public health interventions (e.g. drug control policies).

* Some of these conditions that are mainly acquired when people are hospitalised or in contact with health services might also be considered to be preventable, in the sense that the incidence of these health care-associated infections or health problems might be reduced through greater prevention in health care facilities.

** Drug-related deaths include both illegal and legal drugs.

Section S3. List of ICD-10 codes used in classification of mortality from non-communicable diseases

Source:

World Health Organisation (2014). WHO Global Monitoring Framework on Noncommunicable Diseases: Indicator Definitions and Specifications. Geneva, WHO.

Cause of death	ICD-10 code(s)
Cancer	C00-C97
Cardiovascular disease	I00-I99
Chronic respiratory disease	J30-J98
Diabetes mellitus	E10-E14

Section S4. Additional results

Figure S4.1. Adjusted hazard ratio for all-cause premature mortality, comparing each exposure combination to unexposed population

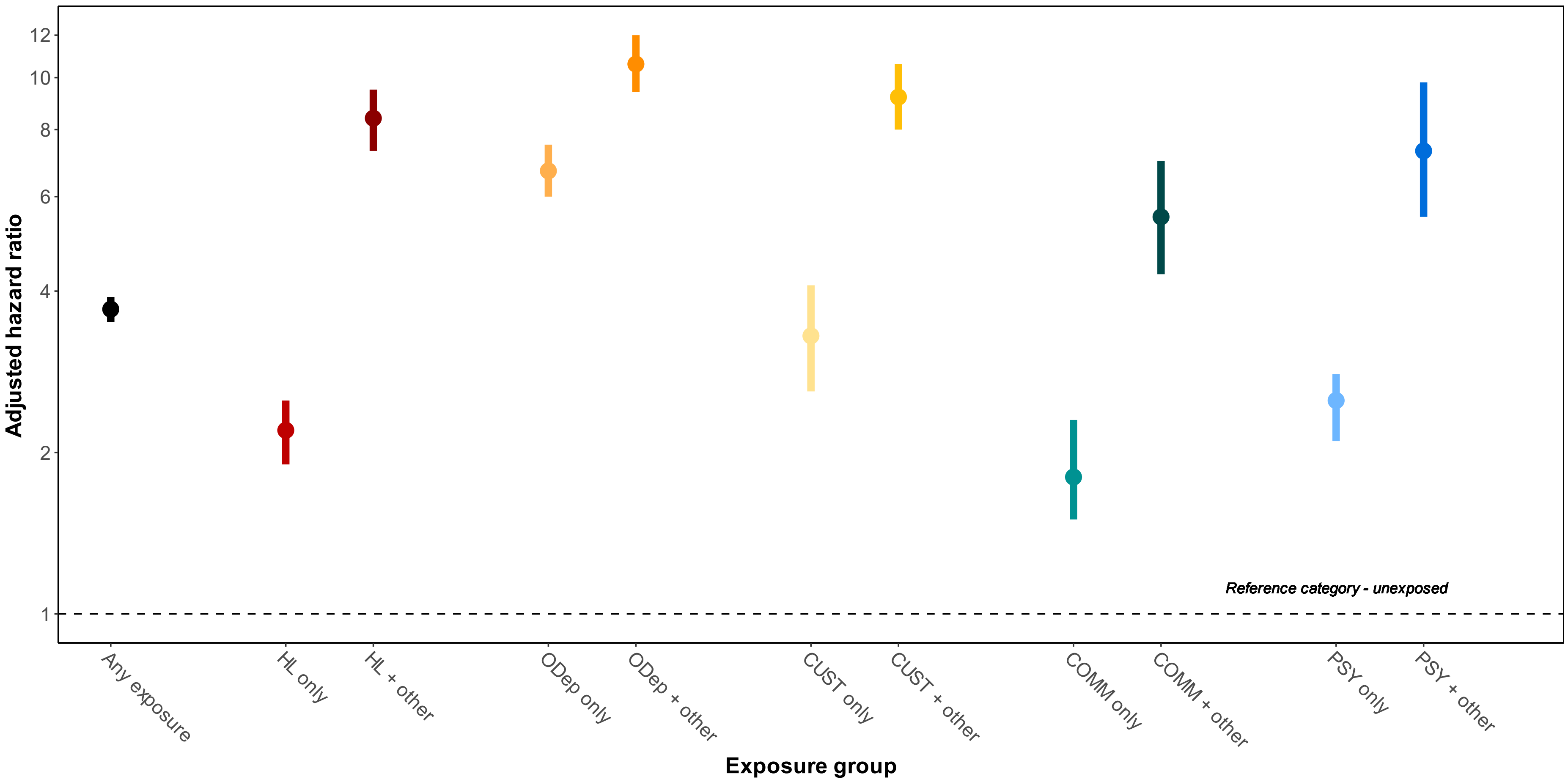

Note that exposure combinations are ordered by frequency of any flag for that exposure.

Table S4.1. Adjusted hazard ratios for all-cause mortality among the cohort by exposure combination, incorporating interaction between exposure and year of follow-up

	**Adjusted hazard ratio for all-cause mortality* (95% confidence interval)**				
	**Year 1** (01/04/2014 – 31/03/2015)	**Year 2** (01/04/2015 – 31/03/2016)	**Year 3** (01/04/2016 – 31/03/2017)	**Year 4** (01/04/2017 – 31/03/2018)	**Year 5** (01/04/2018 – 31/03/2019)
**Exposure status**					
Unexposed	1.0 (reference)	1.0 (reference)	1.0 (reference)	1.0 (reference)	1.0 (reference)
Any exposure	4.5 (4.0 – 5.0)	3.2 (2.8 – 3.7)	3.9 (3.4 – 4.4)	3.7 (3.2 – 4.1)	3.1 (2.7 – 3.5)
**Homelessness**					
HL only	2.5 (1.9 – 3.3)	2.1 (1.5 – 2.9)	2.6 (2.0 – 3.4)	2.1 (1.6 – 2.8)	1.7 (1.2 – 2.3)
HL + other	11.1 (8.7 – 14.1)	5.9 (4.2 – 8.3)	10.0 (7.7 – 12.8)	7.4 (5.6 – 9.8)	7.4 (5.6 – 9.8)
**Opioid dependence**					
ODep only	8.5 (6.8 – 10.6)	6.1 (4.6 – 8.0)	5.6 (4.3 – 7.3)	7.4 (5.9 – 9.2)	6.1 (4.7 – 7.8)
ODep + other	14.5 (11.6 – 18.2)	8.4 (6.2 – 11.4)	11.6 (9.0 – 14.8)	9.7 (7.5 – 12.60)	9.0 (6.9 – 11.7)
**Justice – custodial**					
CUST only	5.6 (3.8 – 8.1)	2.8 (1.6 – 4.9)	2.9 (1.8 – 4.8)	2.8 (1.7 – 4.6)	2.5 (1.5 – 4.2)
CUST + other	13.1 (10.1 – 16.9)	7.5 (5.3 – 10.8)	8.9 (6.5 – 12.2)	7.7 (5.6 – 10.6)	8.8 (6.5 – 11.9)
**Justice – community**					
COMM only	2.2 (1.4 – 3.5)	2.2 (1.3 – 3.6)	2.3 (1.5 – 3.7)	1.6 (1.0 – 2.7)	1.0 (0.5 – 1.9)
COMM + other	7.1 (4.4 – 11.3.)	5.8 (3.3 – 10.0)	6.7 (4.1 – 10.8)	4.9 (2.9 – 8.3)	3.6 (1.9 – 6.6)
**Psychosis**					
PSY only	2.2 (1.6 – 3.0)	2.1 (1.5 – 3.0)	2.9 (2.2 – 3.9)	2.8 (2.1 – 3.6)	2.1 (1.6 – 2.9)
PSY + other	10.8 (6.4 – 18.3)	5.2 (2.3 – 11.6)	4.6 (2.0 – 10.3)	8.1 (4.6 – 14.4)	7.7 (4.2 – 14.0)

Exposure combinations are ordered by frequency of any flag for that exposure. *Unexposed population as reference group. Adjusted for age, gender, SIMD quintile, year of follow-up, and interaction term between year of follow-up and exposure combination.

Figure S4.2. Adjusted hazard ratio for all-cause mortality among those with any versus no exposures of interest, accounting for interaction between exposure and year of follow-up

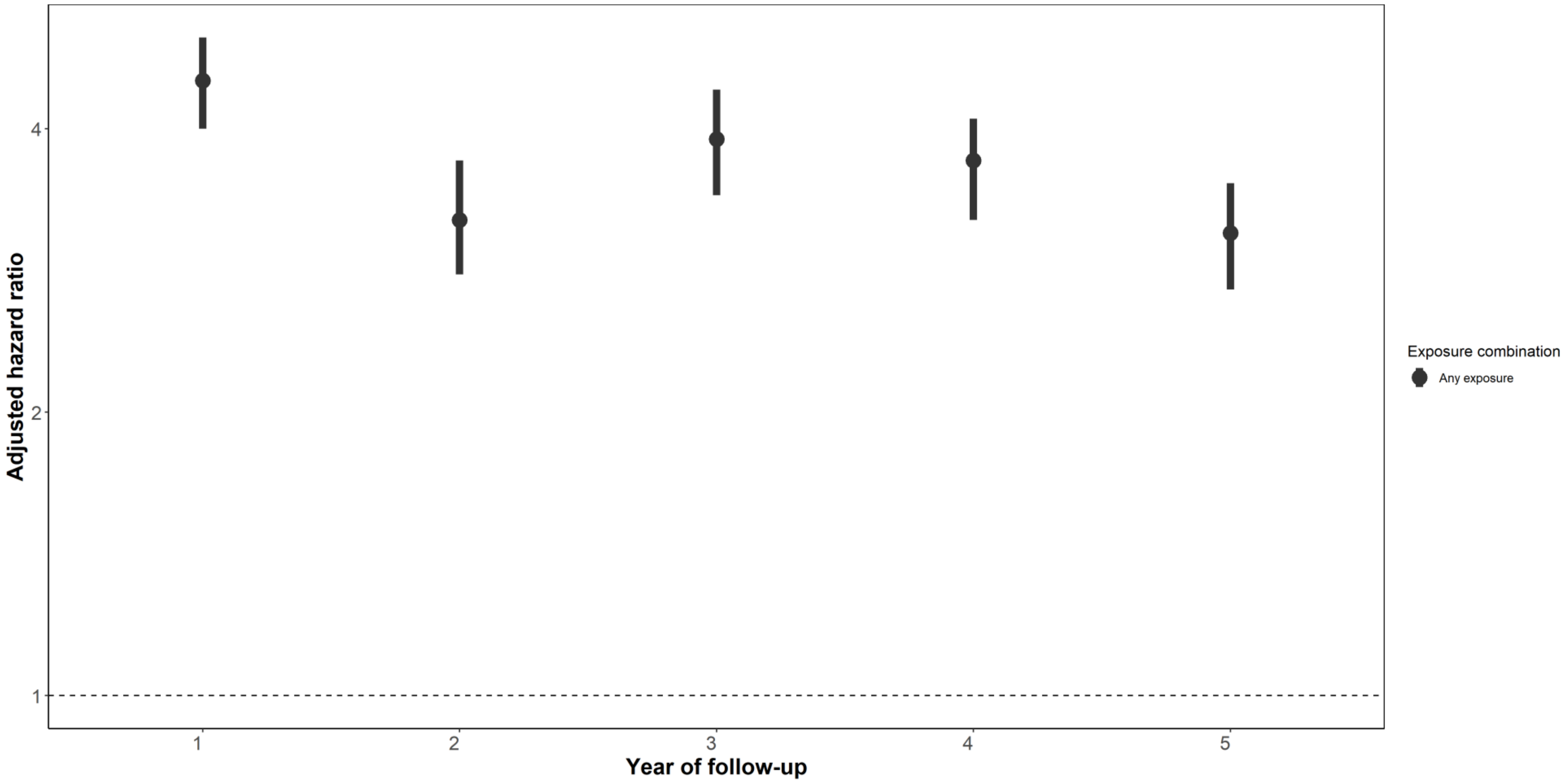

Unexposed population as reference group. Adjusted for age, gender, SIMD quintile, year of follow-up, and interaction term between year of follow-up and exposure combination.

Figure S4.3. Adjusted hazard ratio for all-cause mortality among those with homelessness alone or in combination with other exposures compared to the unexposed population, accounting for interaction between exposure and year of follow-up

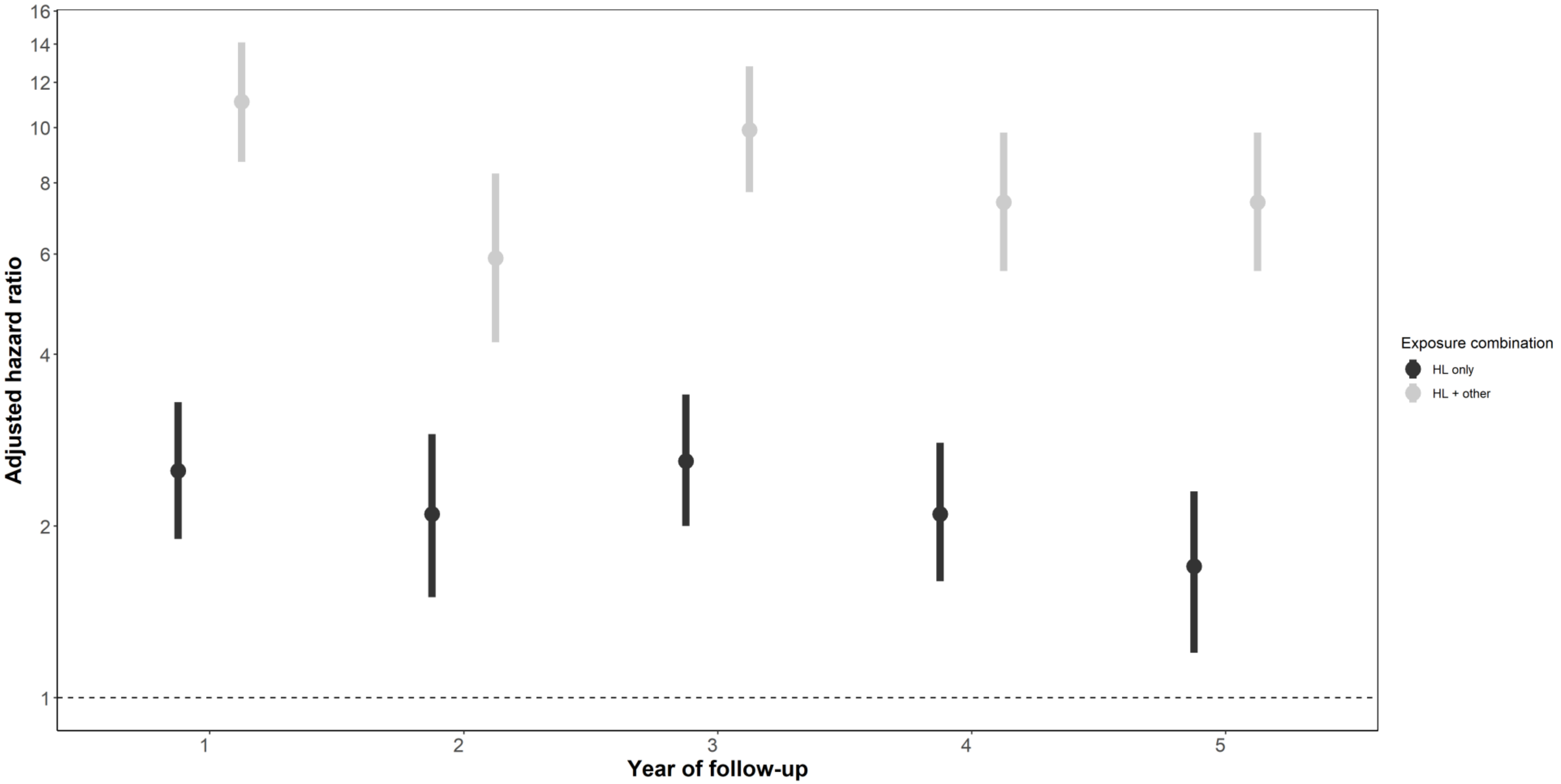

Unexposed population as reference group. Adjusted for age, gender, SIMD quintile, year of follow-up, and interaction term between year of follow-up and exposure combination.

Figure S4.4. Adjusted hazard ratio for all-cause mortality among those with opioid dependence alone or in combination with other exposures compared to the unexposed population, accounting for interaction between exposure and year of follow-up

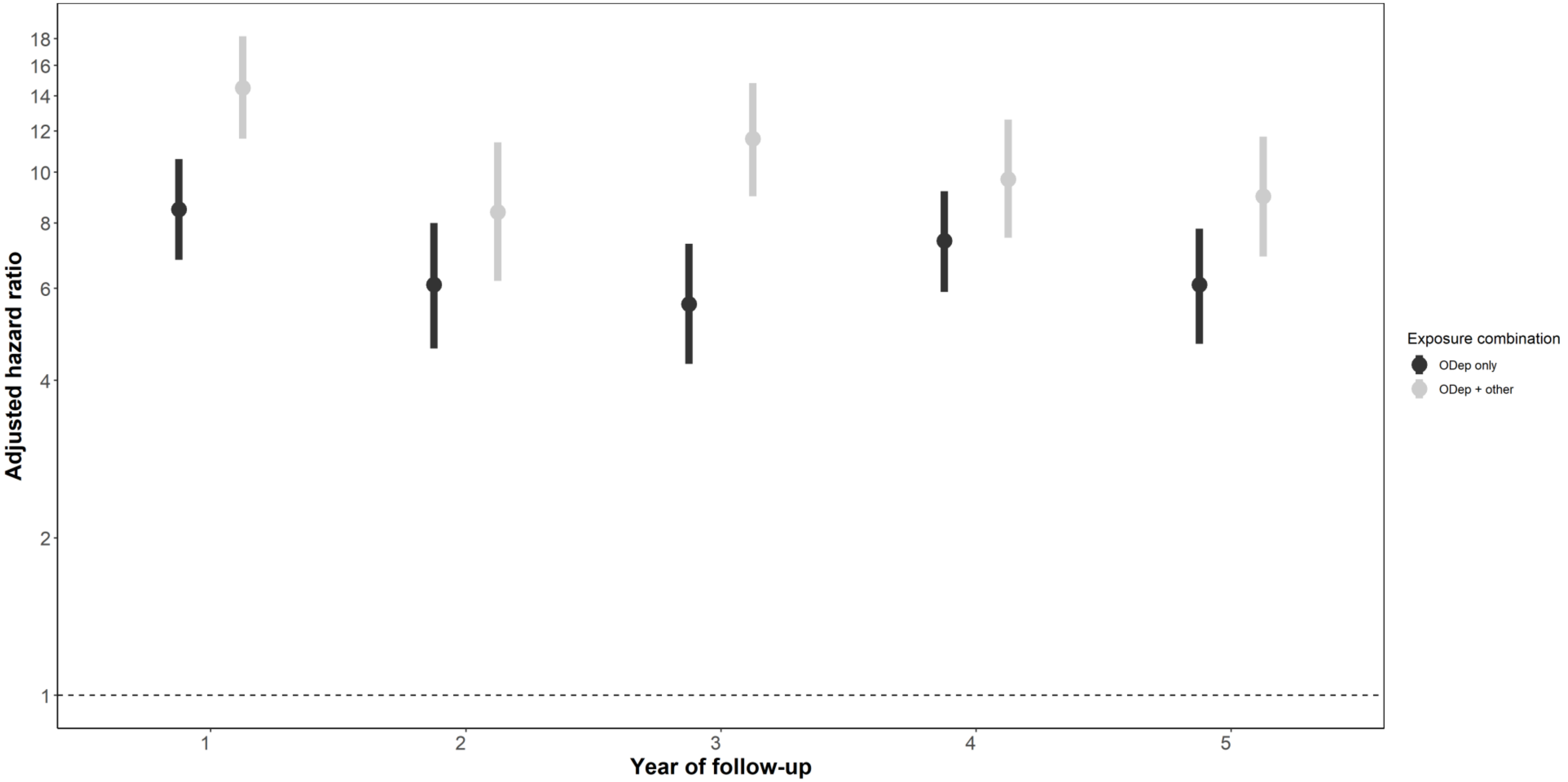

Unexposed population as reference group. Adjusted for age, gender, SIMD quintile, year of follow-up, and interaction term between year of follow-up and exposure combination.

Figure S4.5. Adjusted hazard ratio for all-cause mortality among those with imprisonment alone or in combination with other exposures compared to the unexposed population, accounting for interaction between exposure and year of follow-up

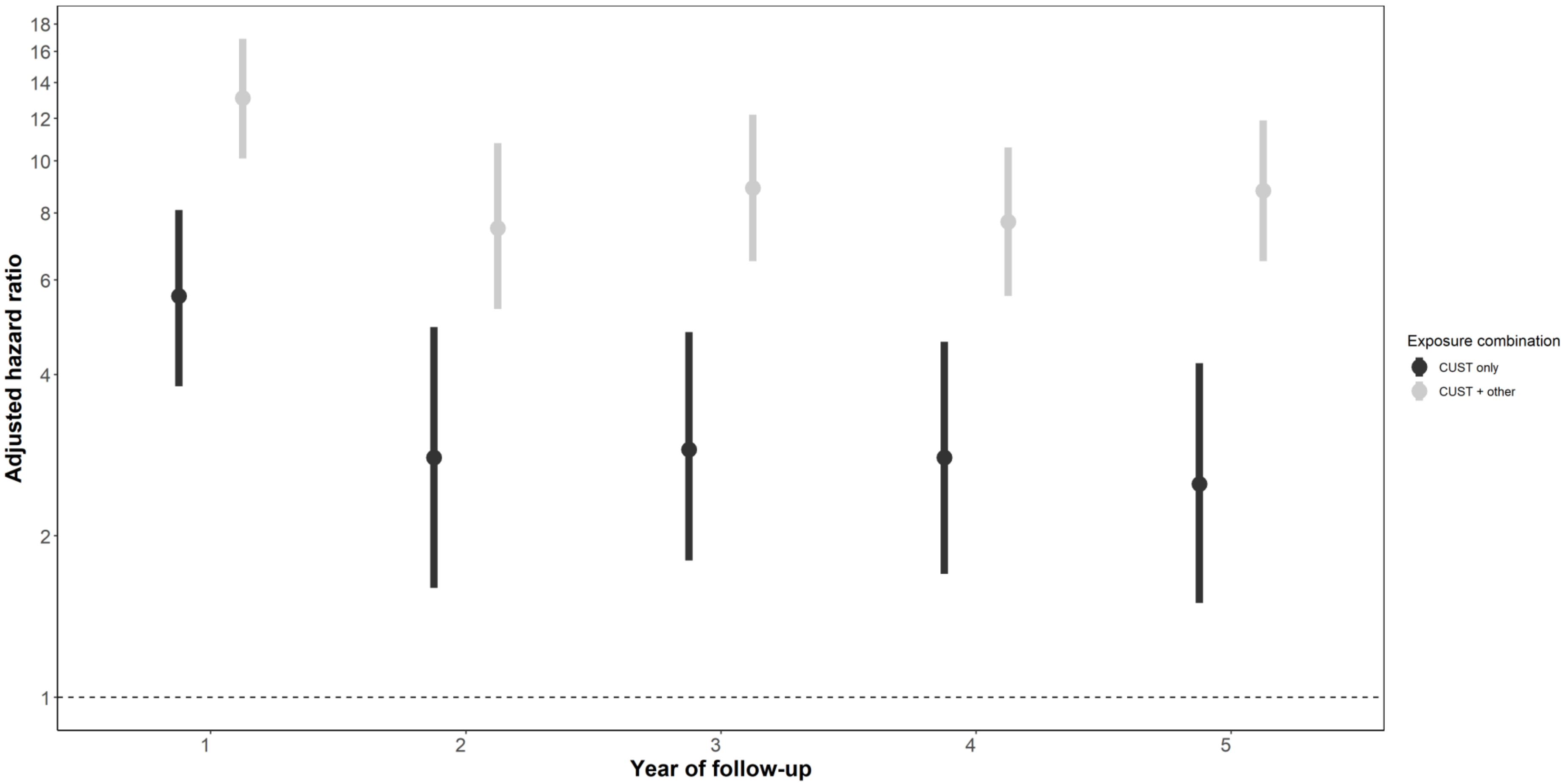

Unexposed population as reference group. Adjusted for age, gender, SIMD quintile, year of follow-up, and interaction term between year of follow-up and exposure combination.

Figure S4.6. Adjusted hazard ratio for all-cause mortality among those with community justice involvement alone or in combination with other exposures compared to the unexposed population, accounting for interaction between exposure and year of follow-up

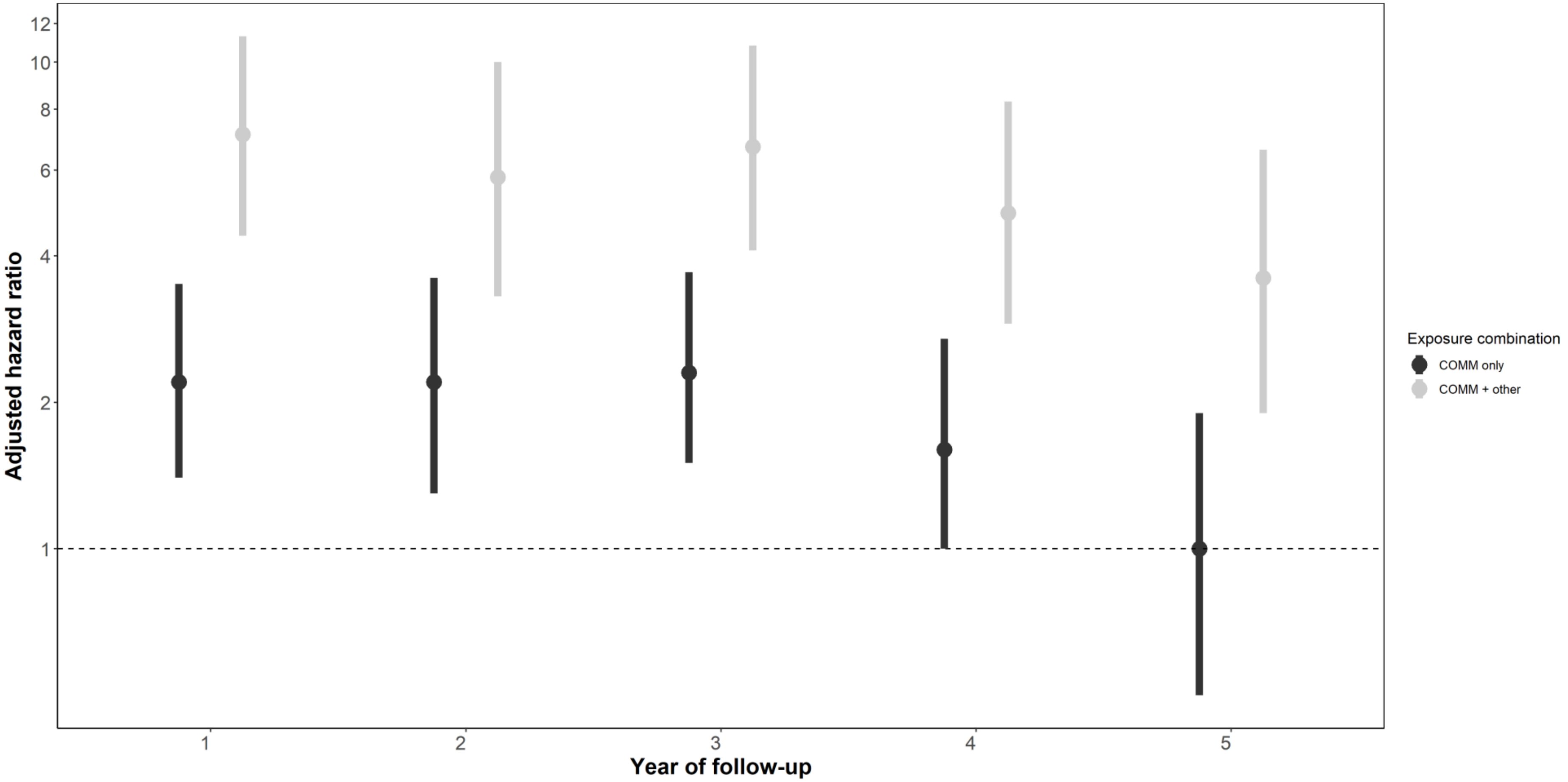

Unexposed population as reference group. Adjusted for age, gender, SIMD quintile, year of follow-up, and interaction term between year of follow-up and exposure combination.

Figure S4.7. Adjusted hazard ratio for all-cause mortality among those with psychosis alone or in combination with other exposures compared to the unexposed population, accounting for interaction between exposure and year of follow-up

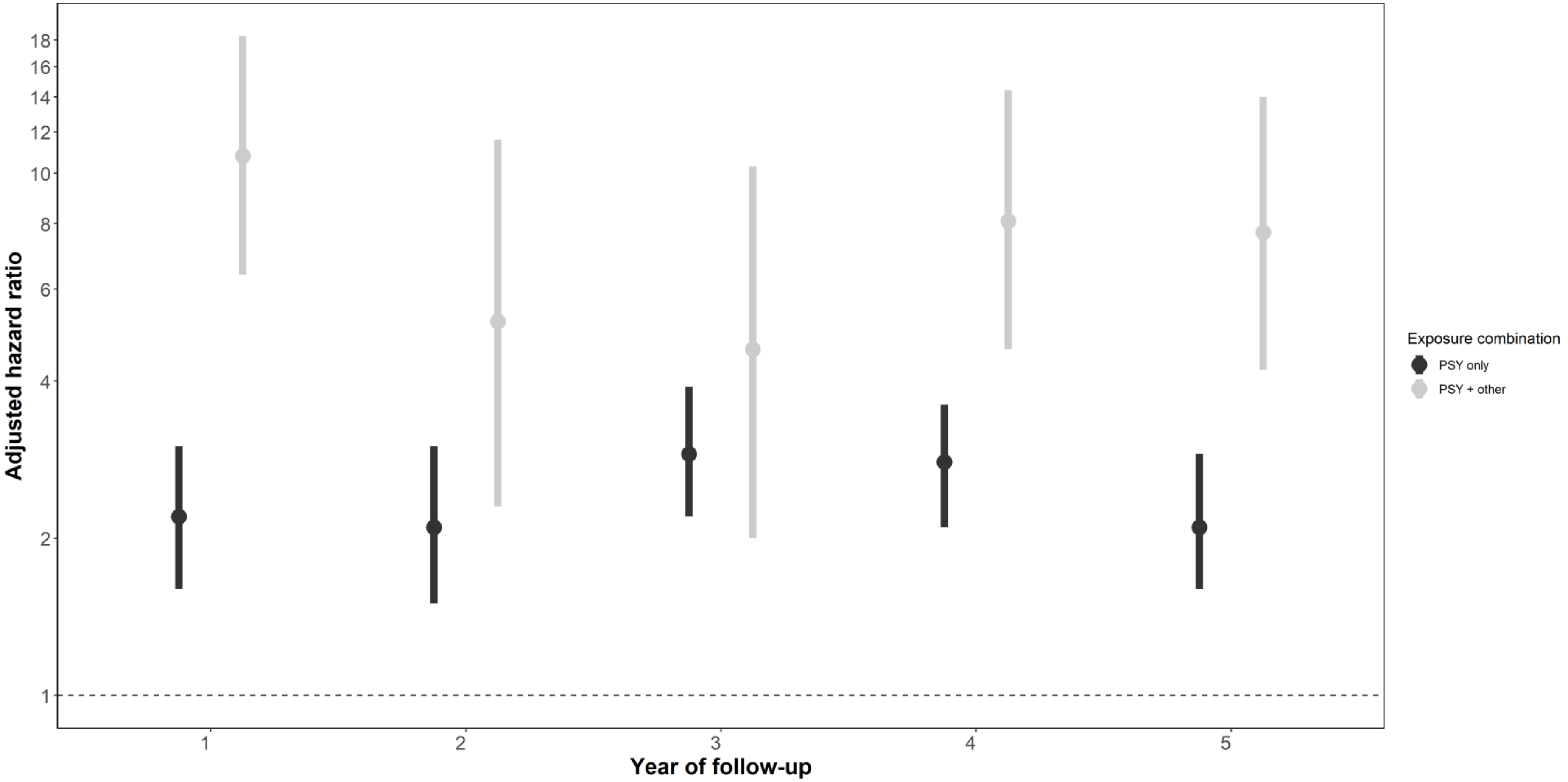

Unexposed population as reference group. Adjusted for age, gender, SIMD quintile, year of follow-up, and interaction term between year of follow-up and exposure combination.

Figure S4.8. Adjusted hazard ratios for all-cause mortality by exposure combination and period of analysis, comparing the exposure period (01/04/2010 – 31/03/2014) to the outcome period used for primary analyses of mortality (31/03/2019)

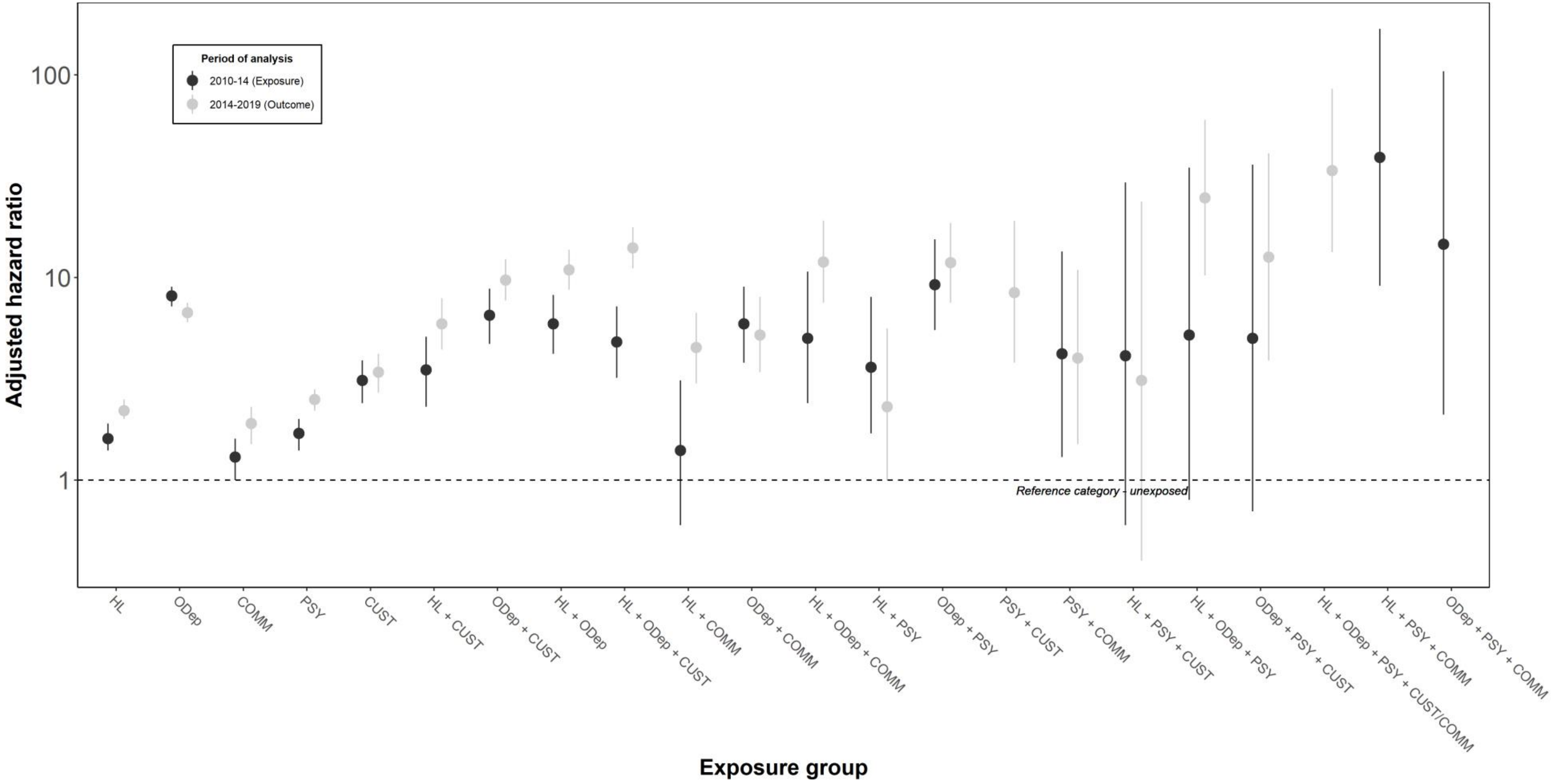

Ordered by frequency of mutually exclusive categories.

Unexposed population as reference group. Adjusted for age, gender, SIMD quintile, year of follow-upyear of follow-up..

Table S4.2. Adjusted hazard ratios for all-cause mortality by exposure combination and period of analysis, comparing the exposure period (01/04/2010 – 31/03/2014) to the outcome period used for primary analyses of mortality (01/04/2014 – 31/03/2019)

Exposures of interest	Number of individuals in cohort	Adjusted hazard ratios (95% CI)	
		Exposure period (01/04/2010 – 31/03/2014)	Outcome period (01/04/2014 – 31/03/2019)
Unexposed	508,541	1.00	1.00
Homelessness (HL) only	9,463	1.6 (1.4 -1 .9)	2.2 (2.0 – 2.5)
Opioid dependence (ODep) only	4,123	8.1 (7.2 – 9.0)	6.7 (6.0 – 7.5)
Justice – community (COMM) only	3,338	1.3 (1.0 – 1.8)	1.9 (1.5 – 2.3)
Psychosis (PSY)	3,255	1.7 (1.4 – 2.0)	2.5 (2.2 – 2.8)
Justice – custodial (CUST) only	2,755	3.1 (2.4 – 3.9)	3.4 (2.7 – 4.2)
HL + CUST	994	3.5 (2.3 – 5.1)	5.9 (4.4 – 7.9)
ODep + CUST	846	6.5 (4.7 – 8.8)	9.7 (7.7 – 12.3)
HL + ODep	820	5.9 (4.2 – 8.2)	10.9 (8.7 – 13.7)
HL + ODep + CUST	780	4.8 (3.2 – 7.2)	14.0 (11.1 – 17.7)
HL + COMM	574	1.4 (0.6 – 3.1)	4.5 (3.0 – 6.7)
ODep + COMM	433	5.9 (3.8 – 9.0)	5.2 (3.4 – 8.0)
HL + ODep + COMM	195	5.0 (2.4 – 10.7)	11.9 (7.5 – 18.6)
HL + PSY	159	3.6 (1.7 – 8.0)	2.3 (1.0 – 5.6)
ODep + PSY	135	9.2 (5.5 – 15.4)	11.8 (7.5 – 18.6)
PSY + CUST	61	-	8.4 (3.8 – 19.0)
PSY + COMM	56	4.2 (1.3 – 13.4)	4.0 (1.5 – 10.9)
HL + PSY + CUST	35	4.1 (0.6 – 29.4)	3.1 (0.4 – 23.7)
HL + PSY + ODep	26	5.2 (0.8 – 34.8)	24.7 (10.2 – 59.8)
ODep + PSY + CUST	25	5.0 (0.7 – 36.0)	12.6 (3.9 – 41.1)
HL + ODep + PSY + any justice*	19	-	33.7 (13.3 – 85.5)
HL + PSY + COMM	10	39.1 (9.1 – 168.3)	-
ODep + PSY + COMM	10	14.6 (2.1 – 104.1)	-

Exposure combinations are ordered by frequency of mutually exclusive categories.

Table S4.3. Effect measure modification for all-cause mortality by additional exposures, on additive and multiplicative scales

		Additional exposures			
		No		Yes	
		N deaths/total	Adjusted hazard ratio (95% CI)	N deaths/total	Adjusted hazard ratio (95% CI)
**Any homelessness**	**No**	10,103/508,541	*1.0 (reference)*	870/15,037	3.8 (3.5 – 4.0)
	**Yes**	241/9,463	2.2 (1.9-2.5)	270/3,612	8.2 (7.3 – 9.4)
	aHR for HL within strata of other exposures	2.3 (2.0–2.6)		1.8 (1.5-2.0)	
	Measure of effect modification on additive scale (RERI); 1.2<br>Measure of effect modification on multiplicative scale: 1.00 (0.8 – 1.2, p=0.975)				
**Any opioid dependence**	**No**	10,103/508,541	*1.0 (reference)*	724/20,700	2.5 (2.3-2.7)
	**Yes**	347/4,123	6.7 (6.0-7.5)	310/3,289	10.6 (9.4-11.9)
	aHR for ODep within strata of other exposures	7.0 (6.2 – 7.8)		3.3 (2.9 – 3.8)	
	Measure of effect modification on additive scale (RERI); 0.4<br>Measure of effect modification on multiplicative scale (95% CI);: 0.6 (0.5-0.7), p=<0.001				
**Any justice involvement - custodial**	No	10,103/508,541	*1.0 (reference)*	1,074/22,600	3.3 (3.1-3.6)
	Yes	88/2,755	3.3 (2.6-4.1)	219/2,757	9.1 (7.9-10.5)
	aHR for CUST within strata of other exposures	3.4 (2.8-4.3)		2.2 (1.9-2.5)	
	Measure of effect modification on additive scale (RERI); 1.5<br>Measure of effect modification on multiplicative scale (95% CI); *P*: 0.8 (0.6-1.1), p<0.001				
**Any justice involvement - community**	No	10,103/508,541	*1.0 (reference)*	1,232/23,493	3.9 (3.6 – 4.1)
	Yes	77/3,338	1.8 (1.4-2.3)	72/1,281	5.4 (4.3-6.9)
	aHR for COMM within strata of other exposures	1.9 (1.5-2.4)		1.2 (0.9-1.5)	
	Measure of effect modification on additive scale (RERI); -1.3<br>Measure of effect modification on multiplicative scale (95% CI); *P*: 0.8 (0.6-1.1), p=0.136				
**Any psychosis**	No	10,103/508,541	*1.0 (reference)*	1,105/24,321	4.0 (3.8-4.3)
	Yes	227/3,255	2.5 (2.1-2.8)	49/536	7.3 (5.4-9.7)
	aHR for PSY within strata of other exposures	*2.5 (2.2-2.8)*		1.8 (1.3-2.4)	
	Measure of effect modification on additive scale (RERI); -0.2<br>Measure of effect modification on multiplicative scale (95% CI); *P*: 0.7 (0.5-1.0)				

aHR; adjusted hazard ratio. aHR is adjusted for age, gender, Scottish Index of Multiple Deprivation, and year of follow-up.

Table S4.4. Number and proportion of avoidable deaths (of which preventable and treatable) by exposure combination

		Number of deaths (% of total deaths, by row)		
	**All-cause**	**Avoidable**	**Preventable***	**Treatable***
Total population	11,484	8,121 (70.7)	5,901.5 (51.4)	2,219.5 (19.3)
No exposures of interest	10,103	7,017 (69.5)	4,964.5 (49.1)	2,052.5 (20.3)
Any exposure of interest	1,381	1,104 (79.9)	937 (67.8)	167 (12.1)
**Any homelessness (HL)**	**511**	**412 (80.6)**	**357 (69.9)**	**55 (10.8)**
HL only	241	185 (76.8)	146 (60.6)	39 (16.2)
HL + other exposures	270	227 (84.1)	211 (78.1)	16 (5.9)
**Any opioid dependence (ODep)**	**657**	**551 (83.9)**	**501.5 (76.3)**	**49.5 (7.5)**
ODep only	347	286 (82.4)	250.5 (72.2)	35.5 (10.2)
ODep + other exposures	310	265 (85.5)	250 (81.0)	14 (4.5)
**Any prison experience (CUST)**	**307**	**257 (83.7)**	**234 (76.2)**	**23 (7.5)**
CUST only	88	74 (84.1)	63 (71.6)	11 (12.5)
CUST + other exposures	219	183 (83.6)	171 (78.1)	12 (5.5)
**Any justice involvement without imprisonment (COMM)**	**149**	**128 (85.9)**	**107.5 (72.1)**	**20.5 (13.8)**
COMM only	77	66 (85.7)	53 (68.8)	13 (16.9)
COMM + other exposures	72	62 (86.1)	54.5 (75.7)	7.5 (10.4)
**Any psychosis (PSY)**	**276**	**195 (70.7)**	**147.5 (53.4)**	**47.5 (17.2)**
PSY only	227	154 (67.8)	109.5 (48.2)	44.5 (19.6)
PSY + other exposures	49	41 (83.7)	38 (77.6)	3 (6.1)

Note that exposure combinations are ordered by frequency of any flag for that exposure.

*OECD/Eurostat definition of preventable and treatable deaths includes some causes which are classified as 50% preventable, 50% treatable (and therefore 100% avoidable). Figures in these columns may therefore include non-integers reflecting the preventable and/or treatable fraction within a given population group.

Table S4.5. Mortality from avoidable causes, by exposure combination and age group

	Number of individuals at start of follow-up (person-years at risk)	Age-stratified rate per 100,000 person-years (95% CI)				Crude HR* (95% CI)	Adjusted HR** (95% CI)
		18-29 yrs	30-44 yrs	45-59 yrs	60-74 yrs		
**Exposure status**							
Unexposed	508,541 (2,367,741.8)	7.6 (5.7 – 10.1)	53.6 (48.6 – 59.1)	322.1 (308.6 – 336.3)	1,355.3 (1,316.2 – 1,395.5)	1.0	1.0
Any exposure	28,112 (134,354.0)	152.9 (115.5 – 202.3)	663.4 (601.3 – 731.9)	1,401.1 (1,284.3 – 1,528.5)	2,450.2 (2,087.9 – 2,875.4)	2.8 (2.6 – 3.0)	4.1 (3.9 – 4.4)
**Homelessness**							
HL only	9,463 (45,335.5)	32.4 (13.5 – 77.9)	250.7 (187.8 – 334.7)	1,1002.3 (822.3 – 1,221.8)	2,201.3 (1,458.0 – 2,802.2)	1.4 (1.2 – 1.6)	2.4 (2.1 – 2.8)
HL + other	3,612 (170,041.3)	437.5 (272.0 – 703.7)	1,306.3 (1,094.7 – 1,558.8)	2,355.5 (1,899.6 – 2,920.9)	1,853.4 (695.6 – 4,938.3)	4.5 (3.9 – 5.1)	9.7 (8.4 – 11.2)
**Opioid dependence**							
ODep only	4,123 (19,631.2)	410.3 (102.6 – 1,640.7)	1,048.8 (880.2 – 1,249.8)	2,081.4 (1,768.8 – 2,449.4)	5,399.1 (3,197.6 – 9,116.2)	4.9 (4.4 – 5.5)	7.8 (6.9 – 8.8)
ODep + other	3,289 (15,431.7)	905.2 (471.0 – 1,739.6)	1,420.3 (1,210.9 – 1,665.9)	2,754.7 (2,273.0 – 3,338.4)	3,276.8 (461.6 – 23,262.3)	5.8 (5.1 – 6.6)	12.6 (11.1 – 14.4)
**Justice – custodial**							
CUST only	2,755 (13,137.2)	207.9 (115.1 – 375.3)	414.4 (270.2 – 635.6)	1,275.7 (897.2 – 1,814.0)	3,168.1 (1,754.5 – 5,720.7)	1.9 (1.5 – 2.4)	3.8 (3.0 – 4.8)
CUST + other	2,757 (12,948.2)	672.8 (418.3 – 1,082.3)	1,262.6 (1,032.6 – 1,543.8)	2,541.8 (2,014.3 – 3,207.4)	0.0 (-)	4.8 (4.1 – 5.5)	10.5 (9.1 – 12.3)
**Justice – community**							
COMM only	3,338 (16,302.2)	145.8 (72.9 – 291.6)	310.8 (198.2 – 487.2)	618.2 (417.7 – 914.8)	2,128.3 (1,260.5 – 3,593.6)	1.4 (1.1 – 1.7)	2.2 (1.7 – 2.8)
COMM + other	1,281 (6,154.0)	296.9 (111.4 – 791.2)	916.8 (641.0 – 1,311.3)	1,599.2 (1,062.7 – 2,406.6)	5,173.2 (2,153.2 – 12,428.8)	3.4 (2.6 – 4.4)	6.6 (5.1 – 8.6)
**Psychosis**							
PSY only	3,255 (15,491.5)	94.1 (13.3 – 667.9)	298.8 (173.5 – 514.5)	979.9 (776.6 – 1,236.5)	2,436.5 (1,924.4 – 3,084.9)	3.4 (2.9 – 3.9)	2.4 (2.0 – 2.8)
PSY + other	536 (2,533.4)	921.6 (297.2 – 2,857.5)	1,263.7 (774.2 – 2,062.7)	2,248.8 (1,450.8 – 3,485.6)	3,818.6 (955.0 – 15,268.5)	5.5 (4.0 – 7.4)	8.6 (6.3 – 11.8)

Exposure combinations are ordered by frequency of any flag for that exposure. *Unexposed population as reference group. ** Unexposed population as reference group. Adjusted for age, gender, SIMD quintile, and calendar time.

Table S4.6. Number and proportion of deaths from non-communicable diseases by exposure combination

	Number (% of total deaths, by row)	
	**All-cause**	**NCD deaths**
Total population	11,484	8,080 (70.4)
No exposures of interest	10,103	7,539 (74.6)
Any exposure of interest	1,381	541 (39.2)
**Any homelessness (HL)**	**511**	**179 (35.0)**
HL only	241	126 (52.3)
HL + other exposures	270	53 (19.6)
**Any opioid dependence (ODep)**	**657**	**181 (27.5)**
ODep only	347	116 (33.4)
ODep + other exposures	310	65 (21.0)
**Any justice involvement - custodial (CUST)**	**307**	**67 (21.8)**
CUST only	88	25 (28.4)
CUST + other exposures	219	42 (19.2)
**Any justice involvement - community (COMM)**	**149**	**57 (38.3)**
COMM only	77	35 (45.5)
COMM + other exposures	72	22 (30.6)
**Any psychosis (PSY)**	**276**	**163 (59.1)**
PSY only	227	147 (64.8)
PSY + other exposures	49	16 (32.7)

Exposure combinations are ordered by frequency of any flag for that exposure.

Table S4.7. Mortality from non-communicable diseases (NCD), by exposure combination

	Number of individuals at start of follow-up (person-years at risk)	Number of NCD deaths (% of all deaths)	Age-stratified rate per 100,000 person-years (95% CI)				Crude HR* (95% CI)	Adjusted HR** (95% CI)
			18-29 yrs	30-44 yrs	45-59 yrs	60-74 yrs		
**Exposure status**								
Unexposed	508,541 (2,367,741.8)	7,539 (74.6)	5.4 (3.8 – 7.5)	42.1 (37.8 – 47.0)	336.6 (322.7 – 351.0)	1,515.4 (1,474.0 – 1,557.9)	1.0	1.0
Any exposure	28,112 (134,354.0)	541 (39.2)	34.3 (19.0 – 62.0)	150.0 (122.0 – 184.4)	809.7 (722.1 – 907.9)	2,401.2 (2,042.8 – 2,822.5)	1.3 (1.2 – 1.4)	2.2 (2.0 – 2.4)
**Homelessness**								
HL only	9,463 (45,335.5)	126 (52.3)	6.5 (0.9 – 46.0)	103.6 (66.1 – 162.3)	726.2 (575.5 – 916.4)	1,965.1 (1,411.0 – 2,737.0)	0.9 (0.7 – 1.0)	1.7 (1.4 – 2.0)
HL + other	3,612 (170,041.3)	53 (19.6)	77.2 (25.0 – 239.4)	127.4 (72.4 – 224.4)	936.5 (665.8 – 1,317.3)	2,316.8 (964.3 – 5,566.2)	1.0 (0.7 – 1.3)	2.7 (2.0 – 3.5)
**Opioid dependence**								
ODep only	4,123 (19,631.2)	116 (33.4)	205.2 (28.9 – 1,456.5)	268.5 (189.9 – 379.7)	1,019.2 (807.7 – 1,286.1)	4,627.8 (2,628.2 – 8,148.8)	1.9 (1.5 – 2.2)	3.6 (3.0 – 4.3)
ODep + other	3,289 (15,431.7)	65 (21.0)	201.1 (50.3 – 804.3)	206.9 (136.3 – 314.3)	1,059.5 (777.2 – 1,444.4)	3,276.8 (461.6 – 23,262.3)	1.3 (1.0 – 1.7)	3.7 (2.9 – 4.8)
**Justice – custodial**								
CUST only	2,755 (13,137.2)	25 (28.4)	56.7 (18.3 – 175.8)	19.7 (2.8 – 140.1)	452.7 (250.7 – 817.4)	2,808.1 (1,549.6 – 5,352.8)	0.6 (0.4 – 0.9)	1.4 (0.9 – 2.1)
CUST + other	2,757 (12,948.2)	42 (19.2)	158.3 (59.4 – 421.8)	132.9 (71.5 - 247.0)	1,002.4 (692.1 – 1,451.8)	0.0 (-)	1.0 (0.8 – 1.4)	2.9 (2.1 – 4.0)
**Justice – community**								
COMM only	3,338 (16,302.2)	35 (45.5)	36.5 (9.1 – 145.8)	81.8 (34.0 – 196.5)	420.3 (261.3 – 676.2)	1,672.3 (926.1 – 3,019.6)	0.7 (0.5 – 0.9)	1.2 (0.9 – 1.7)
COMM + other	1,281 (6,154.0)	22 (30.6)	0.0 (-)	152.8 (63.6 – 367.1)	764.9 (423,6 – 1,381.1)	6,207.9 (2,788.9 – 13,818.0)	1.1 (0.7- 1.7)	2.7 (1.8 – 4.1)
**Psychosis**								
PSY only	3,255 (15,491.5)	147 (64.8)	0.0 (-)	229.8 (123.7 – 427.1)	897.1 (703.5 – 1,144.0)	2,542.4 (2,018.1 – 3,203.0)	3.0 (2.5 – 3.5)	2.2 (1.8 – 2.6)
PSY + other	536 (2,533.4)	16 (32.7)	0.0 (-)	315.9 (118.6 – 841.7)	1,124.4 (605.0 – 2,089.7)	3,818.6 (955.0 – 15,268.5)	2.0 (1.2 – 3.2)	3.7 (2.3 – 6.1)

Exposure combinations are ordered by frequency of any flag for that exposure. *Unexposed population as reference group. ** Unexposed population as reference group. Adjusted for age, gender, SIMD quintile, and calendar time

Table S4.8. Years of Potential Life Lost – sum total, mean per decedent, and mean per 100,000 people at risk, by exposure combination

Exposures of interest	Sum total	Mean per decedent (95% CI)	Mean per 100,000 people at risk (95% CI)
Unexposed	114344.9	11.3 (11.1 – 11.5)	22,484.9 (22,354.8 – 22,615.6)
Homelessness (HL) only	5058.1	21.0 (19.6 – 22.4)	53,451.4 (51,987.3 – 54,944.0)
Opioid dependence (ODep) only	8966.5	25.8 (25.1 – 26.6)	217,475.9 (213,008.8 – 222,036.2)
Justice – community (COMM) only	2369.7	24.5 (21.6 – 27.4)	56,572.1 (54,038.0 – 59,171.0)
Psychosis (PSY)	3474.5	15.3 (14.1 – 16.5)	106,742.7 (103,208.3 – 110,337.4)
Justice – custodial (CUST) only	2369.7	26.9 (24.1 – 29.7)	86,014.7 (82,596.6 – 89,560.1)
HL + CUST	1453.7	29.1 (25.7 – 32.4)	146,248.8 (138,854.7 -153,994.4)
ODep + CUST	2142.0	28.6 (27.0 – 30.1)	253,194.3 (242,581.6 – 264,146.0)
HL + ODep	2292.1	28.7 (26.9 – 30.4)	279,527.6 (268,185.1 – 291,194.8)
HL + ODep + CUST	2500.9	31.7 (30.1 – 33.2)	320,622.1 (308.196.5 – 333,459.1)
HL + COMM	600.3	24.0 (19.4 – 28.7)	104,588.6 (96,331.9 – 113,238.5)
ODep + COMM	649.6	28.2 (25.0 – 31.5)	150,026.8 (138,795.5 – 162,112.8)
HL + ODep + COMM	677.1	33.9 (30.6 – 37.1)	347,226.9 (321,516.4 – 374,346.1)
HL + PSY	111.3	22.3 (10.0 – 34.5)	69,976.6 (57,429.8 – 84,078.8)
ODep + PSY	512.8	25.6 (22.7 – 28.6)	379,819.0 (347,824.0 – 41,4351.8)
PSY + CUST	163.2	27.2 (11.4 – 43.0)	267,571.1 (227,765.6 – 311,529.7)
PSY + COMM	88.5	22.1 (6.5 – 37.8)	158,066.9 (127,632.7 – 195,575.3)
HL + PSY + CUST		<3 deaths	
HL + PSY + ODep	142.8	28.6 (19.4 – 37.8)	549,220.8 (463,551.1 – 647,892.1)
ODep + PSY + CUST	82.5	27.5 (24.0 – 31.0)	329,837.1 (260,868.1 -407,134.5)
HL + ODep + PSY + any justice involvement*	163.9	32.8 (22.2 – 43.3)	862,549.1 (736,107.2 – 1,005,839.8)
HL + PSY + COMM		<3 deaths	
ODep + PSY + COMM		<3 deaths	

Exposure combinations are ordered by frequency of mutually exclusive categories. Results omit exposure combinations in which <3 deaths occurred during follow-up.

Figure S4.9. Years of potential life lost, by exposure combination

(a) Mean years of potential life lost per decedent and 95% confidence intervals

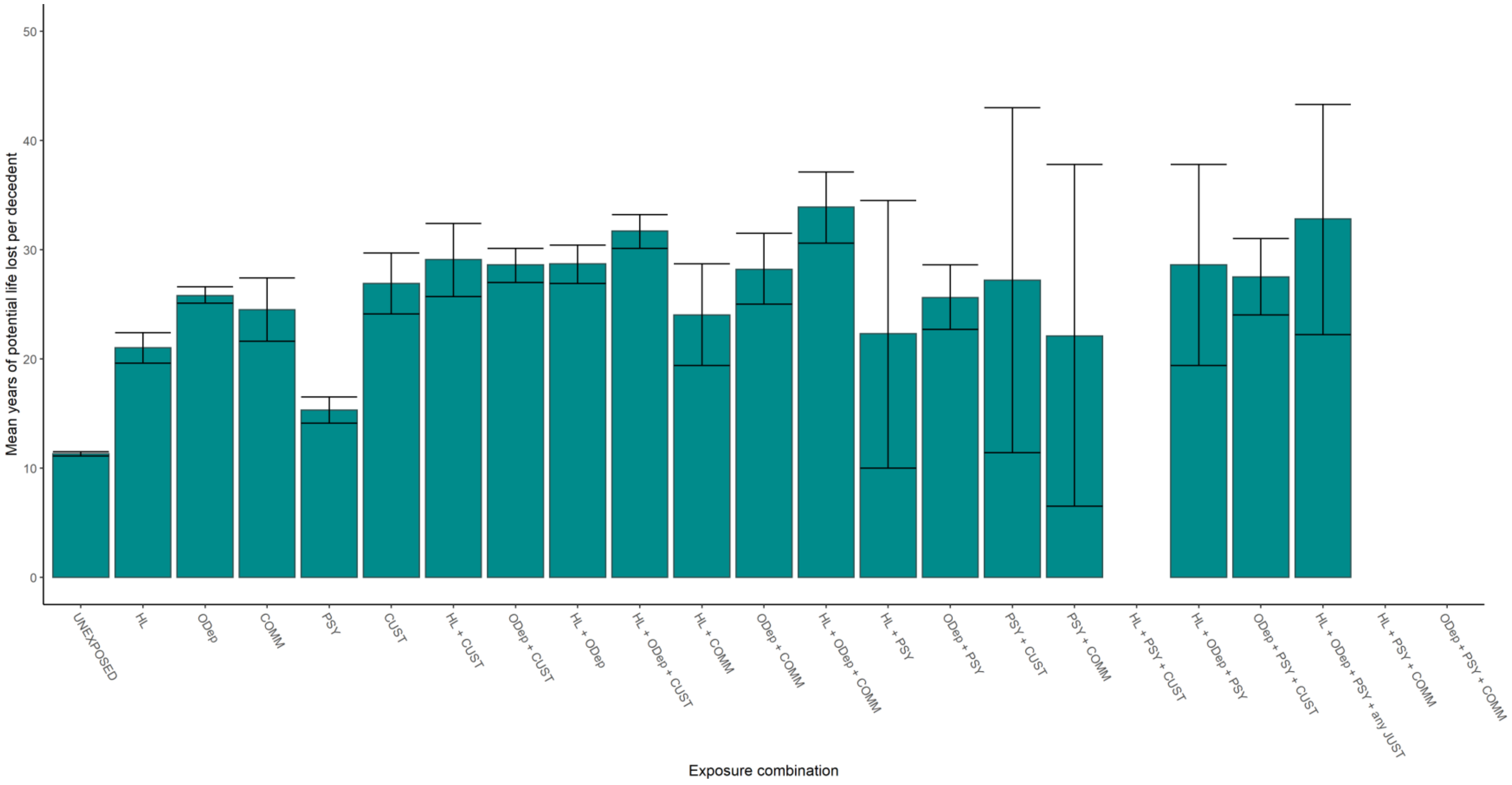

HL – homelessness and housing insecurity; ODep – opioid dependence indicated by receipt of opioid substitution therapy; CUST – imprisonment; COMM – justice involvement in community without imprisonment; PSY – psychosis.

Note that exposure combinations are ordered by frequency of mutually exclusive categories. Results omit exposure combinations in which <3 deaths occurred during follow-up.

(b) Mean years of potential life lost per 100,000 people at risk and 95% confidence intervals

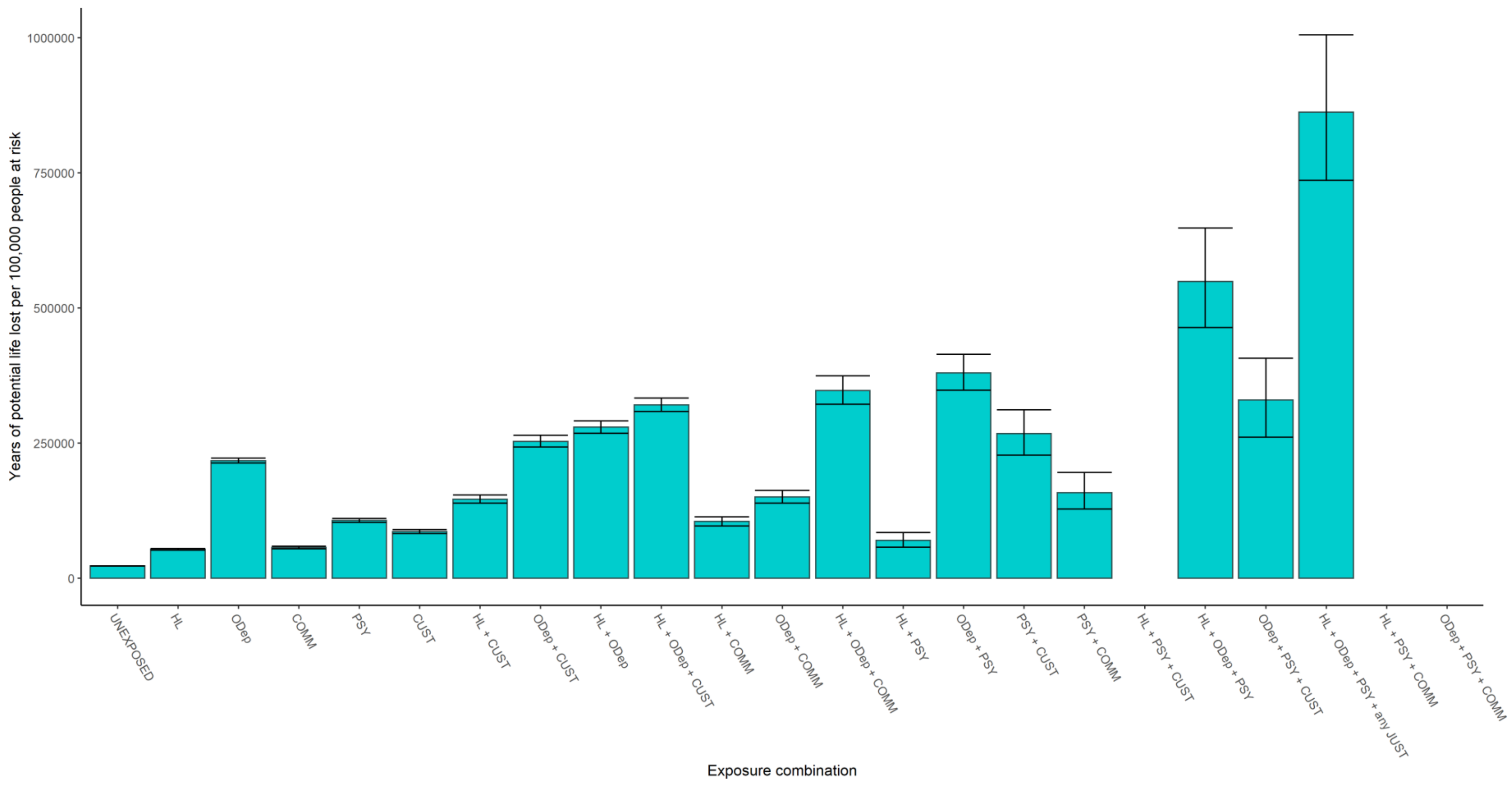

HL – homelessness and housing insecurity; ODep – opioid dependence indicated by receipt of opioid substitution therapy; CUST – imprisonment; COMM – justice involvement in community without imprisonment; PSY – psychosis.

Note that exposure combinations are ordered by frequency of mutually exclusive categories. Results omit exposure combinations in which <3 deaths occurred during follow-up.
